# Drug-Induced Reactivation of Apoptosis Abrogates HIV-1 Infection

**DOI:** 10.1371/journal.pone.0074414

**Published:** 2013-09-23

**Authors:** Hartmut M. Hanauske-Abel, Deepti Saxena, Paul E. Palumbo, Axel-Rainer Hanauske, Augusto D. Luchessi, Tavane D. Cambiaghi, Mainul Hoque, Michael Spino, Darlene D'Alliessi Gandolfi, Debra S. Heller, Sukhwinder Singh, Myung Hee Park, Bernadette M. Cracchiolo, Fernando Tricta, John Connelly, Anthony M. Popowicz, Richard A. Cone, Bart Holland, Tsafi Pe’ery, Michael B. Mathews

**Affiliations:** 1 Department of Biochemistry & Molecular Biology, New Jersey Medical School, Rutgers University, Newark, New Jersey, United States of America; 2 Department of Pediatrics, New Jersey Medical School, Rutgers University, Newark, New Jersey, United States of America; 3 Department of Obstetrics, Gynecology & Women’s Health, New Jersey Medical School, Rutgers University, Newark, New Jersey, United States of America; 4 Oncology Center and Medical Clinic III, Asklepios Clinic St. George, Hamburg, Germany; 5 Leslie Dan Faculty of Pharmacy, University of Toronto, Toronto, Ontario, Canada; 6 ApoPharma Inc., Toronto, Ontario, Canada; 7 Department of Chemistry, Manhattanville College, Purchase, New York, United States of America; 8 Department of Pathology & Laboratory Medicine, New Jersey Medical School, Rutgers University, Newark, New Jersey, United States of America; 9 Oral and Pharyngeal Cancer Branch, National Institute for Dental and Craniofacial Research, Bethesda, Maryland, United States of America; 10 Department of Information Technology, Rockefeller University, New York, New York, United States of America; 11 Department of Biophysics, Johns Hopkins University, Baltimore, Maryland, United States of America; 12 Department of Preventive Medicine & Community Health, New Jersey Medical School, Rutgers University, Newark, New Jersey, United States of America; 13 Department of Medicine, New Jersey Medical School, Rutgers University, Newark, New Jersey, United States of America; George Mason University, United States of America

## Abstract

HIV-1 blocks apoptosis, programmed cell death, an innate defense of cells against viral invasion. However, apoptosis can be selectively reactivated in HIV-infected cells by chemical agents that interfere with HIV-1 gene expression. We studied two globally used medicines, the topical antifungal ciclopirox and the iron chelator deferiprone, for their effect on apoptosis in HIV-infected H9 cells and in peripheral blood mononuclear cells infected with clinical HIV-1 isolates. Both medicines activated apoptosis preferentially in HIV-infected cells, suggesting that the drugs mediate escape from the viral suppression of defensive apoptosis. In infected H9 cells, ciclopirox and deferiprone enhanced mitochondrial membrane depolarization, initiating the intrinsic pathway of apoptosis to execution, as evidenced by caspase-3 activation, poly(ADP-ribose) polymerase proteolysis, DNA degradation, and apoptotic cell morphology. In isolate-infected peripheral blood mononuclear cells, ciclopirox collapsed HIV-1 production to the limit of viral protein and RNA detection. Despite prolonged monotherapy, ciclopirox did not elicit breakthrough. No viral re-emergence was observed even 12 weeks after drug cessation, suggesting elimination of the proviral reservoir. Tests in mice predictive for cytotoxicity to human epithelia did not detect tissue damage or activation of apoptosis at a ciclopirox concentration that exceeded by orders of magnitude the concentration causing death of infected cells. We infer that ciclopirox and deferiprone act via therapeutic reclamation of apoptotic proficiency (TRAP) in HIV-infected cells and trigger their preferential elimination. Perturbations in viral protein expression suggest that the antiretroviral activity of both drugs stems from their ability to inhibit hydroxylation of cellular proteins essential for apoptosis and for viral infection, exemplified by eIF5A. Our findings identify ciclopirox and deferiprone as prototypes of selectively cytocidal antivirals that eliminate viral infection by destroying infected cells. A drug-based drug discovery program, based on these compounds, is warranted to determine the potential of such agents in clinical trials of HIV-infected patients.

## Introduction

Human immunodeficiency virus type 1 (HIV-1) evades the innate and adaptive responses of the immune system, and exploits both to its advantage. In susceptible cells, HIV-1 establishes infection that resists clearance by all current antiretrovirals. Only rarely and under special circumstances may combination antiretroviral therapy (cART) restrain HIV-1 from re-establishing productive infection upon cART cessation, eliciting post-treatment control [1]. The continued presence of HIV-1 DNA in these patients reaffirms the robust resistance of HIV-1 to clearance by pharmacological means. A major feature of this resistance is HIV-1 interference with the primal cellular defense against viral invasion and takeover, programmed cell death (apoptosis) [2]–[5].

After HIV-1 entry, apoptosis remains functional for a brief period [6]. Marked resistance to pro-apoptotic stimuli occurs in HIV-infected cell lines and cultured primary cells, but not their uninfected counterparts, mediated by retroviral proteins and miRNAs [7]–[10]. In brain and blood, infected monomyelocytic cells are protected against apoptosis [11]. Their stable anti-apoptotic gene expression secures viability as mobile infective units and long-term reservoirs [12]. Only 0.1% of productively infected cells in lymph nodes become apoptotic [13]. Furthermore, HIV-1 re-programs susceptible cells to kill uninfected ‘bystanders’ [9], [13], resulting in extensive apoptosis of HIV-specific cytotoxic lymphocytes [14]. T cell depletion, due to virally promoted apoptotic death of uninfected and eventually of infected cells, is the major cause of immune deficiency [12]–[15].

The prominent role of apoptosis in HIV/AIDS was recognized early [16]–[18], suggesting that inhibitors of apoptosis could be combined with antiretrovirals to preserve immune system function by promoting the survival of infected cells and uninfected ‘bystanders’ [13], [19]. While this suggestion remains viable, the studies reported here support an alternative approach, namely the use of *activators* of apoptosis for the ablation of pathogenic HIV-infected cells that destroy the immune system. In oncology, the intentional ablation of pathogenic cells by interventions that activate apoptosis is widely practiced and a leitmotif in anti-cancer drug development [20]. Therapeutic recruitment of the apoptotic mechanism has also been exploited to control graft-versus-host disease in patients [21], but this strategy has not been well explored in virology.

To test the concept of pro-apoptotic therapy in HIV-1 infection, we examined the activity of two medicines previously shown to inhibit HIV-1 gene expression in cellular models and HIV-1 replication in infected PBMCs cultured *ex vivo* [22], [23]. Ciclopirox (CPX; 6-cyclohexyl-1-hydroxy-4-methylpyridin-2[1H]-one: e.g., Batrafen™) is a well-tolerated topical fungicide in gynecological and dermatological preparations. Deferiprone (DEF; 3-hydroxy-1,2-dimethylpyridin-4(1H)-one: e.g., Ferriprox™) is a systemically active medicinal chelator administered orally to transfusionally iron-overloaded thalassemia patients. If successful, our approach would bypass expensive and high-risk preclinical stages of *de novo* drug development by exploiting the off-target activity of approved, globally available non-HIV medicines to define novel, therapeutically desirable on-target effects directly in humans. Precedents for this drug-based drug discovery approach, which builds on easily overlooked side activities of clinically established medicines, include the development of diuretics and oral antidiabetics from the sulfonamide antibiotics, and of antipsychotics from the antihistamines [24].

CPX and DEF are iron-chelating hydroxypyridinones (HOPOs), classified among the 1,2- and 3,4-HOPOs, respectively [25]. Both drugs were identified as candidate inhibitors of protein hydroxylation by searching drug libraries for structures that fit the stereochemical parameters of the catalytic mechanism proposed for 2-oxoacid-utilizing protein hydroxylases [26]–[33]. CPX, DEF, and mimosine, a veterinarily employed DEF analogue, were confirmed to inhibit protein hydroxylation by these dioxygenases at therapeutically achievable concentrations [34], [35]. The compounds were predicted [36] and confirmed [22], [34], [35], [37], [38] to be inhibitors of the hydroxylation of eukaryotic translation initiation factor 5A (eIF5A) by deoxyhypusine hydroxylase (DOHH). DOHH is an apparent monooxygenase whose active site pocket contains a non-heme iron center essential for activity and inhibition [39], [40]. DOHH forms the distinctive hypusine residue of eIF5A, a cellular protein involved in the control of apoptosis [22], [41]–[48] and the replication of HIV-1 and FIV-1 [22], [23], [49]–[54]. DEF and CPX inhibit HIV-1 gene expression at the level of transcript initiation [23], potentially disrupting viral control over cellular apoptosis, acute infection, and the function of the immune system. Consistent with this hypothesis, DEF triggers apoptosis in a latently HIV-infected cell line after mitogen stimulation, but not in its uninfected parent [22], although the underlying pro-apoptotic mechanism was not established.

Here we address the generality of this antiretroviral pro-apoptotic activity and define its mechanism. Our results show that both CPX and DEF overcome retrovirally-induced resistance to apoptosis and activate apoptosis selectively in a chronically HIV-infected CD4^+^ T cell line. The apoptotic mechanism is triggered through the intrinsic mitochondrial pathway. Prior to apoptosis, CPX suppresses acute infection of primary human cells (peripheral blood mononuclear cells, PBMCs) exposed to patient-isolated HIV-1. Self-sustaining HIV-1 infection in long-term PBMC cultures was effectively cleared by CPX and productive infection did not return after cessation of treatment. Despite its pro-apoptotic activity at low µM concentrations in culture, preferentially in infected cells, topical CPX at mM concentrations did not cause deleterious effects in a mouse model predictive for damage to human tissues. Parallel experiments with DEF led to a double-blinded proof-of-concept trial, to be reported elsewhere (Saxena et al., unpublished data). We propose a novel antiretroviral target, the inhibition of protein hydroxylation, and suggest that CPX and DEF can serve as pioneer drugs for exploratory trials and as leads for chemical optimizations with the goal to obtain selectively cytocidal antivirals.

## Results

### CPX and DEF trigger apoptosis in H9 cells via the intrinsic pathway

An earlier study showed that treatment of HIV-infected H9 cells (H9-HIV) with DEF dramatically reduced virion formation and p24 levels [22]. The drug caused nuclear condensation and enhanced DNA fragmentation in latently infected ACH-2 cells induced by phorbol ester, but not in parental CEM cells [22]. These observations, together with accumulating data on the inhibitory action of both CPX and DEF on eIF5A hydroxylation [35], [38], HIV-1 infection in PBMCs and viral gene expression in model systems [23], suggested that the two drugs might have a common mode of action via the induction of apoptosis in HIV-infected cells.

**Figure 1:**
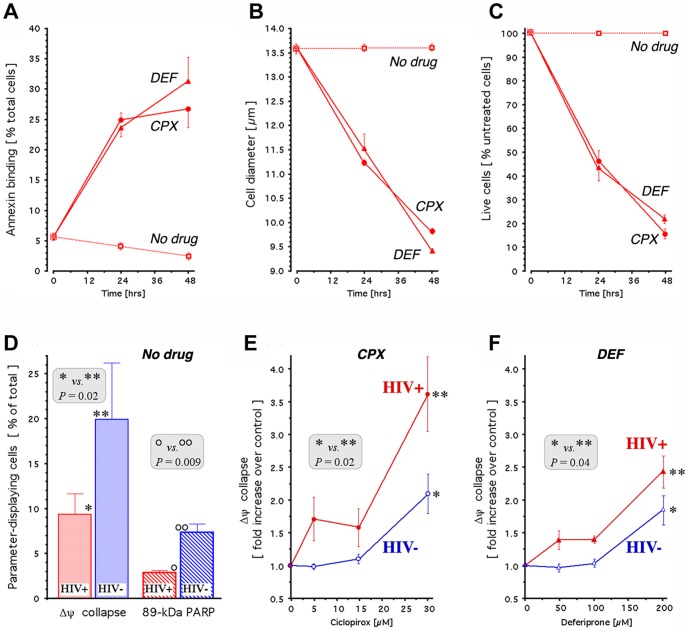
Apoptotic activity of ciclopirox and deferiprone in uninfected and infected H9 cells. **A-C.** Apoptosis in H9-HIV cells treated with 30 µM CPX (circles) or 200 µM DEF (triangles) and in untreated controls (squares). The annexin V- positive and 7-AAD - negative population was quantified by flow cytometry (**A**); cell diameter was quantified by image analysis (**B**); and live cells were quantified by computerized enumeration of trypan blue-stained samples (**C**). **D.** Mitochondrial membrane potential (▵Ψ collapse) and apoptotic proteolysis (89-kDa PARP accumulation) in untreated H9-HIV (red) and H9 (blue) cells. Assays were conducted by flow cytometry 24 hr after plating. Data (mean ± SEM) are calculated as percentage of cell population displaying ▵Ψcollapse or 89-kDa PARP, and *P* values are indicated. **E, F.** Concentration-dependent degradation of mitochondrial membrane potential (▵Ψcollapse) in H9-HIV (red) and H9 (blue), exposed for 24 hr to 30 µM CPX (**E**) or 200 µM DEF (**F**). Results (mean ± SEM) were obtained by flow cytometry using JC-1 and are expressed relative to untreated control cells. *P* values are indicated.

We first determined whether CPX and DEF elicit apoptosis in H9-HIV cells. Annexin-V binding, cell diameter and cell survival were assayed in a time-dependent manner. By 24 hr, drug treatment led to a ∼5-fold increase in the percentage of the 7-amino-actinomycin D (7-AAD)–negative cell population capable of binding annexin-V to exposed membrane phospholipid phosphatidylserine, with little further increase at 48 hr (Fig. 1A). Both drugs caused a decrease in mean cell diameter of ∼15% within 24 hr and ∼30% within 48 hr (Fig. 1B), indicating apoptotic volume decrease (AVD) [55], an early and prerequisite event in apoptosis [56] that reflects cell dismantling into apoptotic bodies [57] and contrasts with the cell volume increase typical of necrosis [58]. Concomitantly, cell survival decreased ∼2 fold at 24 hr and ∼5 fold at 48 hr (Fig. 1C). CPX and DEF exerted similar effects with similar kinetics on these apoptotic indicators, suggesting that both drugs trigger apoptosis in this T lymphocytic cell line chronically infected with HIV-1.

Collapse of the mitochondrial membrane potential, Δψ, is an early event in apoptotic death triggered via the intrinsic pathway, leading to proteolytic activation of initiator and effector caspases including caspase-3 [59]. One consequence of caspase-3 activation is the cleavage of poly (ADP-ribose) polymerase (PARP), resulting in the accumulation of an 89-kDa PARP fragment indicative of nuclear proteolysis [60]. We therefore monitored the Δψ and PARP status of H9 and H9-HIV cells. Flow cytometric analysis showed that both the collapse of Δψ and the cleavage of PARP were attenuated in HIV-H9 cells relative to H9 cells (Fig. 1D). Specifically, Δψ collapse was about half as frequent in HIV-H9 cells as in uninfected H9 cells, and approximately one-third as many cells were positive for PARP cleavage in HIV-H9 cell cultures as in uninfected H9 cultures. These data indicate that apoptosis in H9 cells is triggered via the intrinsic pathway and is attenuated by HIV-1 infection.

### Drug-mediated reversal of resistance to apoptosis in HIV-infected cells

We next compared the effect of the drugs on apoptosis in H9 and H9-HIV cells. Both CPX and DEF increased the collapse of Δψ in a manner that was concentration-dependent and accentuated by viral infection (Fig. 1E,F). Relative to uninfected cells, HIV-infected cells displayed significantly increased collapse of Δψ at the standard drug concentrations used in this study (30 µM CPX; 200 µM DEF). Furthermore, the H9-HIV cultures exhibited enhanced Δψ collapse at lower drug concentrations (5 and 15 µM CPX; 50 and 100 µM DEF) than in uninfected H9 cells (30 µM CPX; 200 µM DEF). Thus, exposure to CPX or DEF counteracted the HIV-mediated reduction of Δψ collapse and rendered infected cells more susceptible than uninfected cells to this early step of apoptosis.

**Figure 2:**
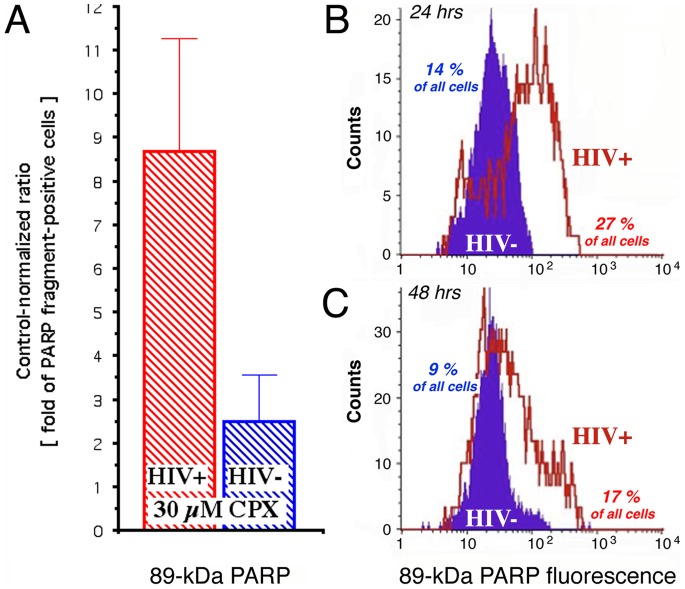
Ciclopirox increases apoptosis preferentially in HIV-infected H9 cells. **A.** Increased formation of the caspase-3–fragmented 89-kDa form of PARP in H9-HIV (red) and uninfected H9 (blue) after 24 hr of treatment with 30 µM CPX. Results (mean ± SEM) are presented as the fold-increase in PARP fragment-positive cells relative to untreated cells. **B, C.** Cell counts over the fluorescence intensity spectrum for 89-kDa PARP reactivity, quantified by flow cytometric single cell analysis after 24 hr (**B**) and 48 hr (**C**) of treatment with 30 µM CPX. Percentages of frag-PARP–positive H9-HIV (red) and uninfected H9 (blue) are calculated.

To determine whether the differential effects of the drugs extend into late apoptosis, we measured PARP fragmentation in H9 and H9-HIV cells. CPX caused an ∼8-fold increase in H9-HIV cells positive for 89-kDa PARP, compared to ∼2 fold in H9 cells (Fig. 2A). By 24 hr, twice as many cells stained positive for 89-kDa PARP in the infected treated cultures as in uninfected treated cultures (27% compared to 14%; Fig. 2B). Furthermore, the fluorescence intensity was approximately one order of magnitude higher in the presence of HIV-1 (Fig. 2B). These differences persisted after 48 hr of CPX treatment (Fig. 2C). DEF gave similar but less pronounced effects (Saxena et al., unpublished data). We conclude that the retroviral suppression of initiation and execution of apoptosis [7]–[13] is reversed by the drugs and transformed into enhanced susceptibility of HIV-infected cells to apoptosis (Fig. 1D vs. Fig. 2A).

### Cellular and viral protein levels during drug-induced apoptosis

**Figure 3:**
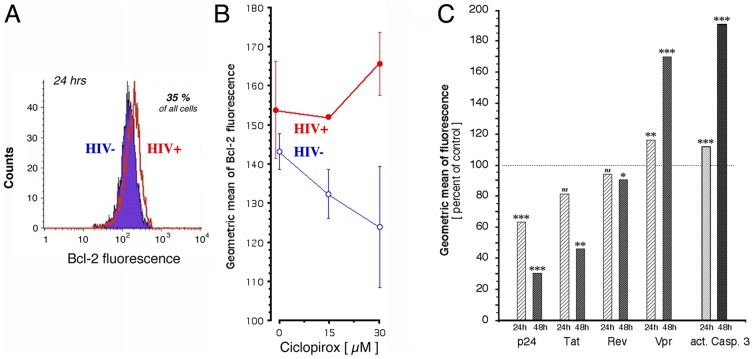
Effects of ciclopirox on cellular and retroviral proteins in H9 cells. **A, B.** Bcl-2 reactivity of cells was quantified by flow cytometry after 24 hr of treatment with CPX. H9-HIV are shown in red, H9 in blue. **A:** Cell counts over the fluorescence intensity spectrum for Bcl-2 reactivity in cells treated with 30 µM CPX. **B:** CPX concentration dependence of Bcl-2 reactivity expressed as the geometric mean of fluorescence (mean ± SEM). **C.** Response of proteins in H9-HIV cells to 30 µM CPX after exposure for 24 hr (hatched bars) and 48 hr (filled bars). Retroviral and cellular proteins were labeled immunocytochemically and quantified in the same sample by flow cytometry. Data are presented as the geometric mean of fluorescence, normalized to time-identical infected untreated controls (100% values at 24/48 hr: p24, 36.2/38.2; Tat, 168.1/141.6; Rev, 7.8/6.4; Vpr, 1.7/1.7; activated caspase-3, 1.3/1.3). *P* values for deviation from respective controls are indicated: * = 0.02; ** ≤ 0.004; *** ≤ 0.0004.

The enhancement of Δψ collapse suggested that the drugs might repress anti-apoptotic proteins that stabilize Δψ, in particular Bcl-2 [61]. CD4^+^ T cells isolated from infected individuals have increased Bcl-2 levels compared to uninfected lymphocytes [62] and several reports implicate HIV-1 Tat in preventing apoptosis in persistently infected cells by inducing Bcl-2 transcription [63]–[65]. Since CPX blocks HIV-1 gene expression [23], we determined Bcl-2 expression in H9 and H9-HIV cells. After treatment with CPX for 24 hr, ∼35% of cells stained positive for Bcl-2, irrespective of infection (Fig. 3A). Although uninfected cells displayed a modest dose-dependent decrease in Bcl-2 content, contrasting with a slight increase in infected cells, neither of these measures achieved statistical significance (Fig. 3B). DEF gave similar results (not shown). These data do not support a role for Bcl-2 suppression in the drug-induced enhancement of apoptosis in H9-HIV cells, in accord with conclusions drawn from a study of other agents that cause apoptosis in HIV-infected cells [8].

Retroviral proteins can control apoptosis. We therefore measured the response of individual retroviral proteins in H9-HIV cells (Fig. 3C) to the shutdown of the HIV-1 promoter by CPX. This drug reduced intracellular p24 by 30% within 24 hr, and by 70% within 48 hr, consistent with results in 293T cells [23]. Intracellular Tat was similarly reduced. Rev was only marginally affected, however, possibly due to its greater stability (the half-life of p24 and Tat is ∼3 hr [66], [67] while that of Rev is at least 16 hr [68]). Paradoxically, the levels of Vpr increased by ∼15% within 24 hr and ∼70% within 48 hr, suggesting a degree of autonomy from transcription-dependent accumulation consistent with previous reports [69], [70]. DEF elicited a similar response, sparing Rev, decreasing p24 and Tat, and increasing Vpr (not shown). The rise in Vpr paralleled that of active caspase-3 (Fig. 3C). Vpr at increased intracellular levels [71] is proapoptotic [72]–[78], and Vpr-driven cell death is characterized by many of the parameters recorded above, such as increased annexin binding [72], changes in cell size and volume [74], Δψ collapse [75], PARP cleavage [76], and DNA fragmentation [77]. By contrast, intracellular Tat displays antiapoptotic activity [63], [65], conducive to establishment of infection and latency [79]. Although Tat and Vpr can each display either anti- or proapoptotic activities depending on the test system [78], [79], in the context of our study the divergence in their levels (Fig. 3C) suggests a functional imbalance between these viral controllers of apoptosis that contributes to the drug-induced apoptotic death of HIV-infected cells (see Discussion).

### Structure-activity relations

**Figure 4:**
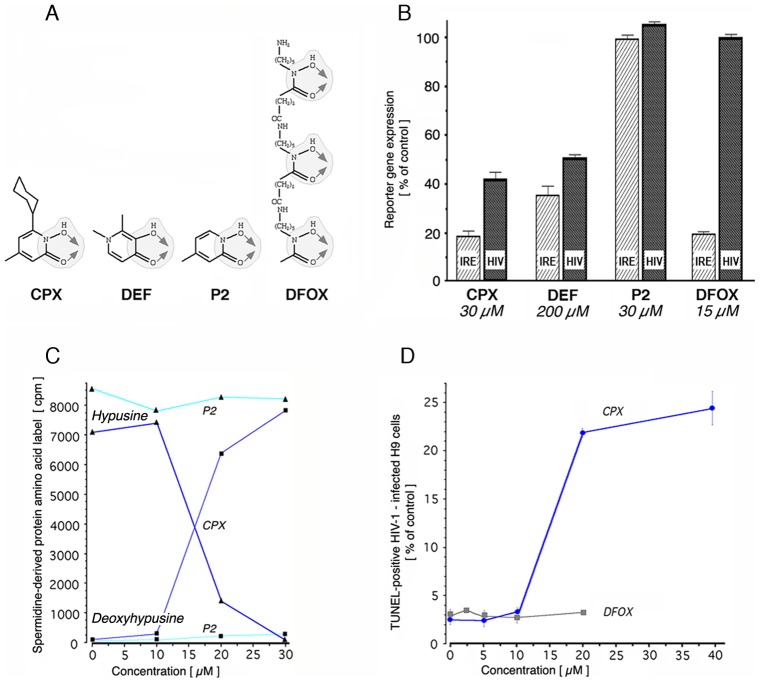
DOHH inhibition, apoptosis, and structure-dependent chelation of intracellular iron. **A**. Covalent structures of the medicinal chelators DFOX and DEF, and of the antifungal agent CPX and its chelation homolog Agent P2. DFOX, CPX, and Agent P2 interact with iron via a hydroxyurea-like hydroxamate moiety that is similar to the chelating domain of DEF (shaded). Arrows indicate this moiety’s uniform bidentate mode of metal binding. DFOX contains three of these moieties and is a hexadentate chelator. **B.** Effect of drugs and Agent P2 on the expression of iron-dependent (IRE; hatched bars) and retrovirally-encoded (HIV; filled bars) gene expression in transfected 293T cells. Results (mean ± standard deviation) are expressed relative to untreated controls. **C.** Inhibition of DOHH activity in H9-HIV by CPX (*blue*), but not by its chelation homolog Agent P2 (*cyan*). *Triangles*, peptide-bound hypusine; *squares*, peptide-bound deoxyhypusine. **D.** Induction of apoptosis by CPX and by DFOX. H9-HIV were treated for 24 hr and then assayed by flow cytometry using TUNEL. Results are expressed as percentage of cells that are TUNEL-positive (± SEM).

Both CPX and DEF are hydroxypyridinones with vicinally positioned oxygen atoms that mediate bidentate interaction with metal ions, in particular Fe^3+^ (Fig. 4A) [25]. To evaluate the effect of iron-chelating drugs on the intracellular iron pool and on HIV-1 gene expression, we exploited transient expression assays in human 293T cells. Intracellular iron was assayed using a construct in which a physiological iron-responsive element (IRE) controls luciferase expression [80]. This approach is highly specific for iron, due to the metal selectivity of the IRE [81], and provides a functional parameter not captured by commercial fluorescent iron sensors (FIS) [82]. HIV-specific transcription was assayed in parallel using an HIV-1 molecular clone that expresses a luciferase reporter under the direction of the retroviral promoter [23]. CPX and DEF were compared with the medicinal chelator deferoxamine (DFOX) and with the CPX fragment Agent P2, which consists of the metal-binding 1,2-HOPO domain of CPX without its cyclohexyl moiety (Fig. 4A).

CPX and DEF reduced intracellular iron by 80% and ∼60%, respectively (Fig. 4B, hatched bars). DFOX, tested at 15 µM, close to the plateau concentration reported in patients (13.7 µM [83]), also reduced intracellular iron by 80% (Fig. 4B). These IRE-based results for DEF and DFOX are consistent with FIS-based data on the depletion of the intracellular iron pool by either drug [84]–[86]. As expected [23] CPX and DEF decreased HIV-1 expression by 40–50% (Fig. 4B, filled bars). DFOX had no effect on HIV-driven gene expression, however, indicating that depletion of chelatable intracellular iron does not translate directly into antiretroviral activity. This conclusion is consistent with the failure of DFOX to suppress HIV-1 gene expression and replication in culture [23], [87] and to prevent disease progression and death in HIV-infected patients [88]. DFOX is an effective inhibitor of iron-containing protein hydroxylases only at supraclinical concentrations [35], [89], [90]. As predicted from its structure, Agent P2 displays a CPX-like ability to form bidentate chelates with Fe^3+^ that produce tris(N-hydroxypyridinone ligand) complexes (Fig. S1A), but it had no effect on either IRE-dependent or HIV-encoded luciferase expression (Fig. 4B) at concentrations equimolar to CPX. Furthermore, Agent P2 failed to inhibit cellular eIF5A hydroxylation even at a concentration at which CPX completely suppressed hydroxylation (Fig. 4C), and its apparent K_i_ for DOHH inhibition *in vitro* was ∼7-times higher than that of CPX (Fig. S1B). Evidently, the cyclohexyl moiety of CPX is required for cell entry, DOHH inhibition, and antiretroviral activity.

The relationship between iron chelation and apoptosis was assessed using the terminal deoxynucleotide transferase dUTP nick end labeling (TUNEL) assay to measure DNA fragmentation. DEF induces this late apoptotic event in HIV-expressing ACH-2 cells [22]. Similarly, CPX triggered DNA fragmentation in H9-HIV cells in a dose-dependent manner with a steep, almost 10-fold increase in TUNEL-positive cells between 10 and 20 µM CPX that leveled off at 40 µM (Fig. 4D). Agent P2 was ineffective (not shown). DFOX did not enhance DNA fragmentation even at 20 µM, which exceeds the peak levels achievable in human plasma [83] (Fig. 4D). We conclude that the antiretroviral activities of CPX and DEF in HIV-infected cells require more than global iron chelation. Specifically, apoptosis and the inhibition of retroviral gene expression by these drugs are not mediated simply via the depletion of extracellular or intracellular ferric ions, or of other metal ions chelated by these drugs.

### Inhibition of acute viral infection and activation of apoptosis in HIV-infected PBMC cultures

To examine the antiretroviral action of CPX in a clinic-derived model, we used PBMCs infected with patient isolates of HIV-1. Naïve freshly isolated PBMCs from a single donor were infected by co-cultivation with HIV-infected, HLA-nonidentical PBMCs at an initial uninfected/infected cell ratio of 10:1. CPX was added and cells were maintained and monitored for six days. Half of each culture supernatant was replenished daily with CPX-containing or CPX-free medium as appropriate. In control cultures, robust infection invariably occurred during this period, achieving p24 and viral RNA copy levels and a p24/viral copy ratio similar to those reported in patients [91].

**Figure 5:**
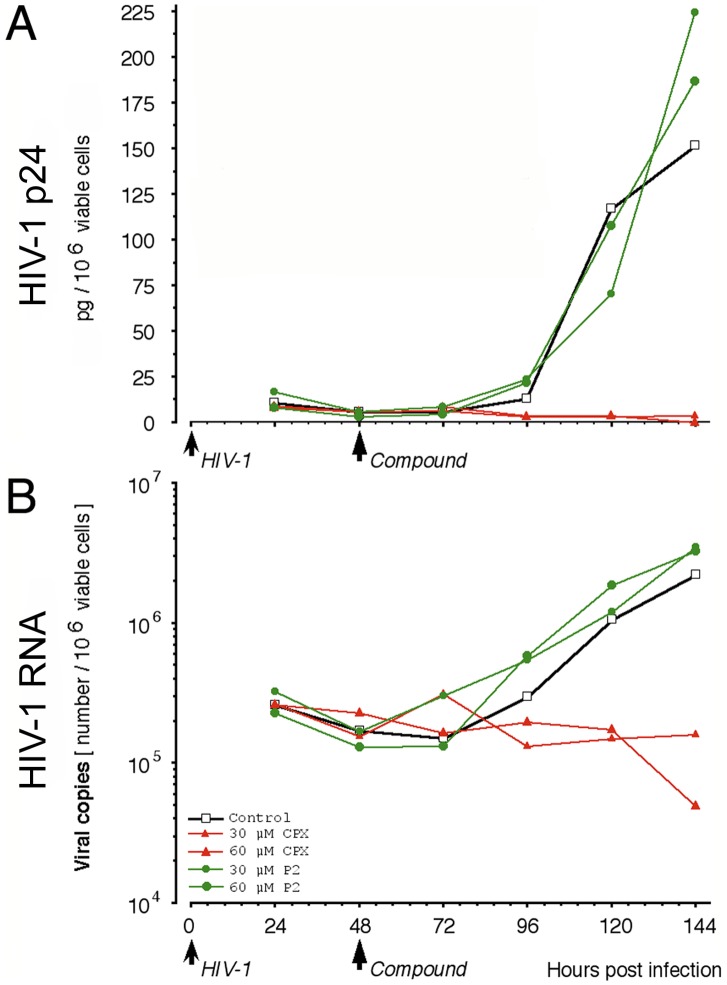
Antiretroviral activity of ciclopirox in slow-onset infection of primary cells. Uninfected PBMCs from a single-donor were infected with isolate #990,135. Cultures were left untreated (open squares) or either CPX (red triangles) or Agent P2 (green circles) was added at 48 hr after plating/inoculation to 30 µM (small symbols) or 60 µM (large symbols). HIV-1 protein (p24; **A**) and copy number (HIV-1 RNA; **B**) were assayed at 24-hr intervals.

The kinetics of infection varied depending on the particular PBMC donor/HIV isolate combination. For the donor/isolate combination shown in Figure 5, p24 formation briskly increased 96 hr after inoculation with HIV-infected PBMCs. Addition of CPX at 48 hr completely inhibited p24 production, as shown previously [23], and doubling the CPX concentration to 60 µM did not further increase this suppressive effect (Fig. 5A). Correspondingly, CPX inhibited the production of HIV-1 RNA (Fig. 5B), indicating that the drug completely suppressed the establishment of productive Infection. Agent P2, the chelation homolog of CPX, did not curtail the accumulation of p24 or HIV-1 RNA (Fig. 5A,B), consistent with its lack of effect on HIV-1 gene expression in H9-HIV cells (Fig. 4B). The delay-insensitivity of the antiretroviral activity of CPX in PBMCs argues against interference with early events such as retroviral binding to cell receptors or fusion with the target cell membrane.

**Figure 6 pone-0074414-g006:**
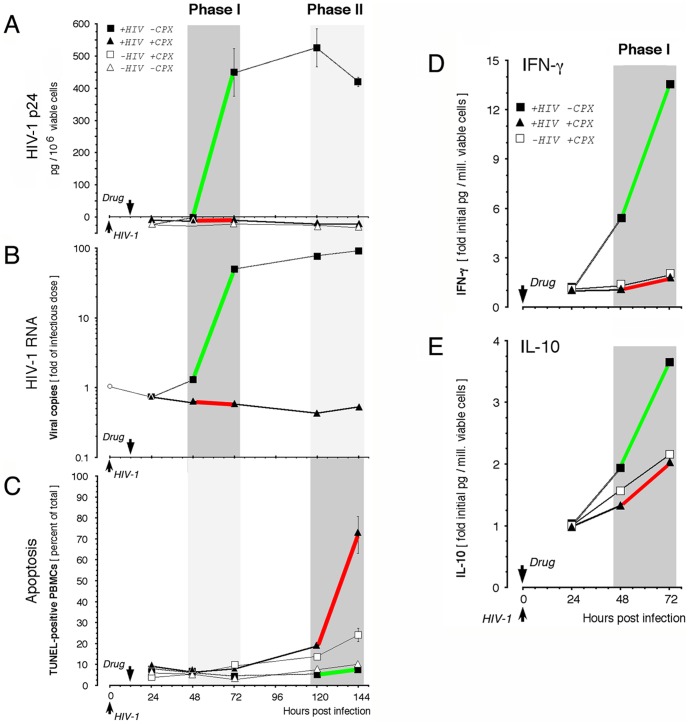
Inhibitory action of ciclopirox in rapid-onset infection of primary cells. **A-C.** Blockade of acute HIV-1 infection and activation of HIV-enhanced apoptosis. Uninfected PBMCs from a single-donor were cultured without infection (open symbols) or were infected with 58,500 copies/ml of HIV-1 isolate #990,010 (filled symbols). After 12 hr, CPX was added to 30 µM (open squares) or cultures were left untreated (triangles). HIV-1 p24 (**A**) and RNA (**B**) were assayed at intervals and apoptotic cells were enumerated by TUNEL (**C**). Active retroviral gene expression occurs in Phase I, preceding suppression of apoptosis in Phase II (green line segments). In CPX-treated infected cultures, retroviral gene expression is inhibited in Phase I and apoptosis is activated in Phase II (red line segments). **D, E.** Response of innate cytokines. Cells were treated as above, except that CPX addition was coincident with infection. IFN-γ (**D**) and IL-10 (**E**) were analyzed during Phase I in the same samples by flow cytometric bead assay. Values are the mean of two independent experiments (initial levels in pg/10^6^ vital cells for HIV-exposed CPX-treated, HIV-exposed untreated, and uninfected CPX-treated cells: 657, 209, and 634 for IFN-γ; 34, 13, and 40 for IL-10).

The induction of apoptosis was analyzed with donor/isolate combinations that displayed rapid emergence of productive infection. In such cultures, the protein and nucleic acid indices of active retroviral infection rose within 72 hr after inoculation with infected PBMCs (Fig. 6A,B). The increases in retroviral p24 and RNA occurred in a synchronous manner (*r* = +0.92, *P* < 0.01). CPX, whether added at the time of inoculation (not shown) or 12 hr later (Fig. 6A,B), inhibited HIV-1 replication. HIV-1 p24 remained undetectable throughout six days of incubation (Fig. 6A), and extracellular HIV-1 RNA did not increase at any time over the initial infectious dose, whereas it increased >100 fold in HIV-exposed untreated cultures (Fig. 6B). Using the TUNEL assay for DNA fragmentation, <10% of cells in infected or uninfected untreated cultures were apoptotic 144 hr after inoculation (Fig. 6C). This population increased to 24.1% (± 3.2%) of cells in CPX-treated uninfected cultures. By contrast, it rose dramatically to 71.8% (± 8.8%) of CPX-treated cells exposed to HIV-1 (*P* = 0.009) (Fig. 6C). Similar to H9 cells (Fig. 2A), CPX roughly doubled the proportion of TUNEL-positive PBMCs in uninfected cultures but caused a ∼10-fold increase in HIV-infected cultures. Apoptosis emerged after 120 hr in treated infected cultures (Fig. 6C; dark gray period), whereas productive infection in untreated PBMC cultures reached a maximum within 72 hr (Fig. 6A,B; dark gray period). Thus, untreated infected PBMCs displayed marked HIV-1 expression early (green segments in Phase I; Fig. 6A-C) and displayed minimal apoptosis subsequently (green segment in Phase II), whereas HIV-exposed CPX-treated PBMCs showed no HIV-1 expression in Phase I but extensive apoptosis in Phase II (red segments; Fig. 6A-C). Similar results were obtained with DEF (Saxena et al., unpublished data). These observations are consistent with the induction of apoptosis in infected cells if the expression of critical HIV-1 genes is blocked.

Cytokines play key roles during HIV-1 infection and predict disease progression [92]. To further assess the action of CPX on HIV-infected primary cells, we measured their ability to launch a cytokine reaction to the virus. We measured the levels of interferon (IFN)-γ and interleukin (IL)-10, since *in vivo* their plasma levels are altered at the earliest point of HIV-1 infection [92], [93]. IFN-γ, a representative Th1 cytokine that activates antiviral defenses, is elevated early in infection and decreases with disease progression [94]. IL-10, a representative Th2 cytokine that limits immune system activity, increases markedly as CD4 counts fall [95], [96]. Both cytokines were measured in infected, CPX-treated or untreated PBMC cultures. In HIV-exposed untreated cultures, the levels of IFN-γ and IL-10 increased at 72 hr by ∼14- and ∼4-fold, respectively (Fig. 6D,E), closely paralleling the rise of viral parameters (Fig. 6A,B; *r* = +0.94, *P* < 0.05). CPX abolished the HIV-induced cell stimulation reflected by IFN-γ and IL-10 synthesis, irrespective of whether the drug was added at the time of inoculation (Fig. 6D,E) or 12 hr later (not shown). IL-6, which is markedly increased in the plasma of HIV-infected persons in a secondary, acute-phase inflammatory response [97], rose by a factor of 5.5 in untreated HIV-exposed PBMC cultures, and by a factor of 1.8 in CPX-treated HIV-exposed cultures (not shown). DEF gave similar results (not shown). In drug-treated infected cultures, the lack of a cytokine response therefore concurred with the inhibition of virological indices (Fig. 6A,B).

We conclude that CPX and DEF block the acute infection of freshly isolated PBMCs by patient isolates of HIV-1 to such an extent that viral production is undetectable and the innate cytokine response is not activated.

### Termination of infection in drug-treated PBMCs

**Figure 7:**
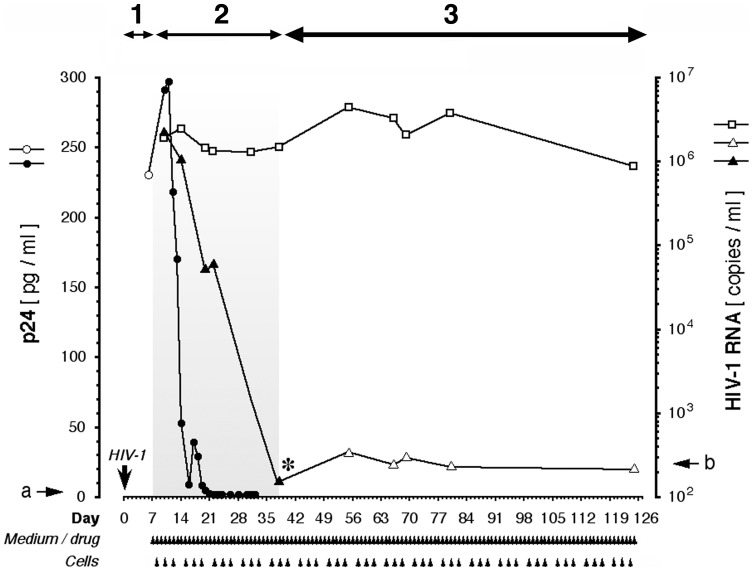
Long-term suppression of HIV-1 infection in PBMC cultures by ciclopirox. Multiple-donor PBMC cultures were infected with isolate #990,010 and replenished with fresh cells and medium as indicated by arrowheads; on each occasion, half of the culture was replaced. After one week (period **1**) to establish infection ex vivo, the culture was treated with 30 µM CPX for one month (period **2**), then the drug was withdrawn (asterisk) and the culture was assayed for viral copy number during three post-treatment months (period **3**) to monitor for re-emerging productive infection. p24 assays: open circle, HIV-exposed untreated cultures; closed circles, HIV-exposed cultures, treated with CPX. HIV-1 RNA assays: open squares, HIV-exposed untreated cultures; closed triangles, HIV-exposed cultures during CPX treatment; open triangles, HIV-exposed cultures after withdrawal of CPX. Arrows **a** and **b** denote the detection limits of the p24 and HIV-1 RNA assays, respectively. Due to the continuous replenishment with freshly isolated uninfected PBMCs, the viability of cultured cells was consistently above 90% as assessed by computerized vital dye exclusion.

We next asked whether CPX can control *established* HIV-1 infection in primary cells. Long-term PBMC cultures were employed as a model for on-going, self-sustaining HIV-1 production. As before, infection was initiated by exposure to patient isolate-infected cells. To emulate the bulk flow of susceptible cells from a generative into an infective compartment that occurs *in vivo*, we followed a replenishment protocol. Freshly isolated uninfected primary cells were infused into the infected cultures at regular intervals during multi-month monitoring of viral parameters. HIV-1 RNA reached the range of 10^6^ copies/ml within a week of patient isolate inoculation (Fig. 7, period 1), and this robust infection was sustained for 4 months (Fig. 7; open squares).

The introduction of CPX on day 7, adjusted daily to maintain a constant level of 30 µM, reduced the virus to the limits of detectability with a week-to-week median decline of ▵log -1.0 (Fig. 7, period 2); individual decline kinetics varied with the donor-isolate combination. Apoptosis parameters (TUNEL and AVD) did not differ from untreated controls (not shown), attributed to the on-going replenishment of the cultures with freshly isolated PBMCs. In the experiment shown here, HIV-1 RNA levels declined during the four week treatment by four orders of magnitude (Fig. 7; closed triangles), whereas the suppression of p24 occurred within 14 days (Fig. 7; closed circles). We attribute the apparent lag in HIV-1 RNA inhibition to the broad dynamic range of the PCR-based RNA assay (note logarithmic scale) compared to the relatively narrow range of the ELISA-based p24 assay (linear scale), and possibly to the packaging of RNA into apoptotic bodies that protect against degradation by RNases [98], [99]. Mathematical modeling (Fig. S2) indicated that the viral RNA level decreases more slowly than the rate calculated for depletion by medium replenishment, arguing against a protocol-related artifactual decline. Evidently, CPX dramatically suppressed viral production in continuous PBMC culture and did not allow viral breakthrough during prolonged monotherapy (≥30 days, Fig. 7). Similar results were obtained with DEF (not shown). By contrast, breakthrough occurs after as few as 20 days of monotherapy with standard antiretrovirals like lamivudine, emtricitabine, zidovudine, nevirapine, or foscarnet [100]–[103], and is prevented only by combining several of these drugs [104]-[107].

To determine whether any productively infected cells survived the suppressive effect of CPX monotherapy, we examined the possibility of viral resurgence following withdrawal of drug. Cultures were maintained for extended post-treatment observation periods and monitored for the re-emergence of HIV-1 RNA (Fig. 7, period 3). Strikingly, after drug cessation (asterisk in Fig. 7) HIV-1 infection did not recur during post-treatment observation periods extending up to 90 days. Similar results were obtained in repeated experiments (not shown). DEF likewise produced off-drug suppression and consistent with apoptotic ablation of infected cells, reduced HIV-1 DNA to the limit of detection (Saxena et al., unpublished data). By contrast, monotherapy with standard antiretrovirals (including zidovudine, lamivudine, nevirapine, delavirdine, loviride, tenofovir, ritonavir, indinavir, saquinavir, stavudine, festinavir, didanosine, or emitricitabine [108], [109]) uniformly fails to delay resurgence of HIV-1 production for more than 3 days off drug, despite an initial report that HIV-1 “became negative” [110]. Marked reduction of HIV-1 RNA without off-drug resurgence requires combination of several of these antiretrovirals [104]–[106].

We conclude that the apparent functional sterilization of HIV-infected primary cultures treated with CPX or DEF correlates with the preferential apoptotic ablation of HIV-infected cells, and thus the destruction of the proviral reservoir, by each of these drugs.

### Lack of apoptosis induction in a murine tissue damage model

In the experiments reported here, CPX and DEF enhanced apoptotic indices in HIV-infected cells and also in uninfected cells, albeit to a significantly lesser degree (Fig. 1E,F; Fig. 6C). We therefore studied the effects of both drugs in model systems predictive of toxicity in humans.

**Figure 8:**
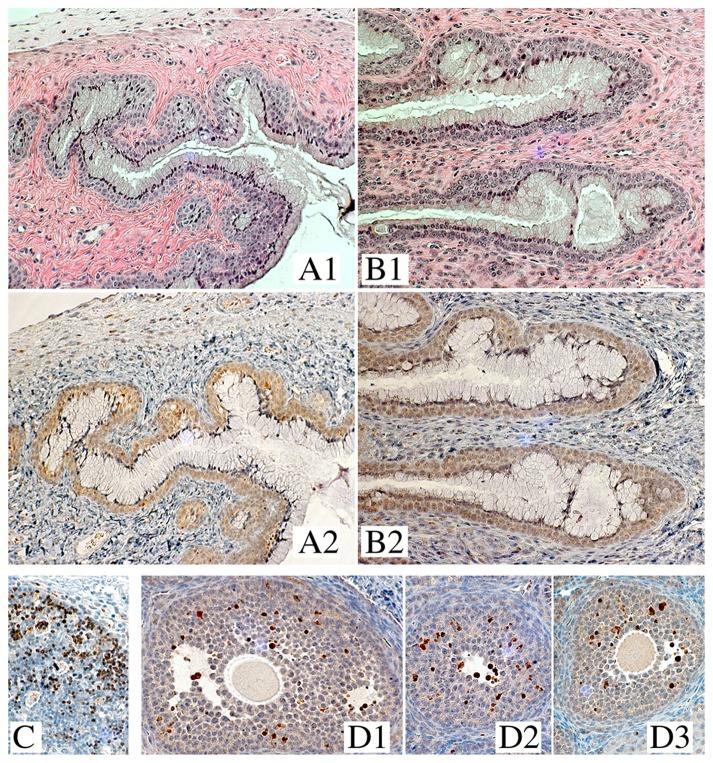
Treatment of mouse vaginal mucosa with ciclopirox. A, B: Histology of vaginal mucosa of medroxyprogesterone-synchronized mice, untreated (A) or intravaginally treated (B) for four consecutive days with the antifungal gynecological formulation of CPX (1% Batrafen Vaginalcrème™, equivalent to 28.8 mM CPX). A1 and B1, stained with hematoxylin-eosin; A2 and B2, stained with anti-active caspase-3. Due to the progestin synchronization of all animals, the vaginal mucosa of untreated (A) and treated (B) animals displays a luminal surface of living cuboidal mucinous cells, overlying uncornified strata of living squamous epithelial cells. C, D: Tissue reactivity to anti-active caspase-3 for two organs known to contain cells undergoing apoptosis, human neonatal thymus (C) [216] and mouse ovary (D1-D3) [217]. Active caspase-3 locates to the nuclei of cortical lymphocytes and folliculogenic cells, respectively, consistent with its established nuclear occurrence [115], and generates a characteristic, punctate staining pattern. Batrafen-treated vaginal mucosa does not display this apoptotic pattern (**B2**), showing instead the faint cytoplasmic reactivity of untreated controls (**A2**). The images of **B2**, evidencing absence of apoptotic cells after vaginal Batrafen exposure, and of **D1**-**D3,** evidencing presence of physiologically apoptotic cells in the ovary, were taken from the same longitudinal cut that sections an animal’s entire reproductive tract.

CPX was evaluated both functionally and histologically for effects on murine vaginal mucosa in a mouse model designed to assess the integrity of genital epithelium via susceptibility to challenges with herpes simplex virus type 2 (HSV-2) [111]–[113]. The gynecological preparation of CPX (1% Batrafen Vaginalcrème™ [Sanofi-Aventis]), whose drug content exceeds the antiretroviral concentration of 30 µM CPX by orders of magnitude, was applied intravaginally. This preparation generates drug levels in human surface tissues that range at 30 µM [114]. Treatment of the mice for four consecutive days did not increase susceptibility to vaginal infection with HSV-2. In the low-dose HSV-2 group, one of the untreated and none of the CPX-treated animals became infected; in the high-dose HSV-2 group, 8 of 10 animals became infected whether CPX-treated or not. This lack of a gross effect on the protective function of the cervicovaginal mucosa was corroborated by histological examination. CPX exposure did not disturb the medroxyprogesterone-induced surface-lining layer of living mucinous cells in these mice, nor did the drug disrupt the underlying layers of squamous epithelial cells (Fig. 8A,B). Notably, CPX did not trigger apoptosis in the epithelial or subepithelial compartments, as assessed by immunohistochemical detection of active caspase-3. This protease locates to the nucleus after induction of apoptosis [115] and therefore reactivity to anti-active caspase-3 displays a typical punctate pattern, highlighting the nuclei of cells undergoing apoptosis in tissue, as shown for human neonatal thymus (Fig. 8C) and mouse ovarian follicles (Fig. 8D). The nuclei of mucinous and squamous epithelial cells in CPX-exposed vaginal mucosa did not react with anti-active caspase-3, and their faint cytoplasmic hue did not differ from untreated mucosa (Fig. 8A2, B2).

DEF was tested in tissue culture for its effect on the trans-epithelial resistance displayed by confluent human ECC-1 cells linked by tight junctions [116]. At 200 µM, a concentration that inhibits HIV-1 replication and ranges at the peak values measured in human plasma [117], DEF did not degrade the barrier function of the monolayer, signifying absence of an apoptotic effect on these uninfected cells (Saxena et al., unpublished data).

In sum, neither CPX nor DEF gave evidence of apoptotic or other toxic effects on uninfected epithelia when applied at or above the concentrations that ablate HIV-infected cells (Figs. 1, 2, 4 and 6) and suppress *de novo* and established infection of PBMCs by viral isolates (Figs. 5–7). The lack of detrimental activity in these models suggests that drug-induced death of uninfected tissue elements *in vivo* is minimal or undetectable, consistent with the clinically established safety record of each medicine.

## Discussion

The drugs investigated here, the antifungal agent CPX and the medicinal chelator DEF, inhibit retroviral gene expression in chronically infected T cells with concomitant activation of the intrinsic pathway of apoptosis, resulting in suppression of acute infection and preferential ablation of infected cells. In isolate-infected cultures of primary cells, CPX produced lasting post-treatment remission, assessed as absence of rebounding replication-competent virus and of resurgent HIV-1 expression. No damage to the uninfected cells of epithelial tissues in mice was detected. In a double-blinded proof-of-concept trial, a one week-course of oral DEF caused an acute zidovudine-like reduction of viral load in individuals who attained the threshold serum level of the drug, defined according to the concentrations required in culture for inhibition of HIV-1 gene expression and induction of apoptosis in infected cells as documented here and elsewhere [22], [23]. The viral load reduction persisted throughout seven weeks of monitoring after cessation of DEF (Saxena et al., unpublished data). These data suggest that medicinal activation of apoptosis in pathogenic infected cells is a viable antiviral strategy, for which we propose the term 'therapeutic reclamation of apoptotic proficiency' (TRAP). Related approaches using recombinant protein constructs rather than small molecules, have been tested against several viruses in cell culture and a mouse model [118], [119]. We infer that the TRAP concept has general applicability for the termination of viral infections.

### Viral *versus* therapeutic control over programmed cell death

Apoptosis is a stereotypical cellular defense against viral takeover. Numerous viruses neutralize this innate initial defense, including HIV-1 [2]–[5], [120]. Several HIV-1 accessory proteins, exemplified by Nef and Vpr, disrupt antiviral resistance [121]. Rapid execution of apoptosis does occur upon entry of HIV-1 into CD4^+^ T cells [6], before the completion of reverse transcription and the Vpr-enhanced expression of anti-apoptotic factors like Nef from unintegrated viral DNA [122]–[124]. However, only a fraction of target T cells eludes the Vpr blockade of anti-viral defenses [125] and is able to mount the innate antiviral suicide response [6]. HIV-1 entry usually leads to expression of viral gene products that interact with cellular components and modulate specific cellular activities, such as apoptosis or cytokine production, to achieve replicative advantage [7], [11], [19], [64], [125]–[127]. As shown here and elsewhere [22], CPX and DEF allow completion of the innate antiviral suicide response by promoting TRAP, thereby overcoming the retrovirally triggered resistance to apoptosis.

TRAP may also underlie the selectively cytocidal, antiretroviral activity of other small molecules. Adenine triggers selective ablation in an HIV-infected CD4^+^ cell line, apparently by changing the association of pro-apoptotic Bax with the mitochondrial adenine nucleotide translocator [128], [129]. Selective induction of apoptosis in HIV-infected lymphocytes was reported for motexafin gadolinium (Xcytrin™) [130], an experimental chemotherapeutic agent that triggers mitochondrially-mediated apoptosis [131] and inhibits the thioredoxin-glutathione system [132], which protects mitochondria from oxidative stress [133]. Imatinib (Gleevec™), an activator of mitochondria-dependent apoptosis used for treatment of chronic myeloid leukemia [134], elicits progressive increase of DNA fragmentation in HIV-infected macrophages, but does not promote apoptotic death of uninfected macrophages [7]. Flavopiridol (Alvocidib™) activates the mitochondrial pathway of apoptosis [135], [136] and causes extensive caspase-3–driven apoptosis in latently infected cell lines, but not their uninfected parents [137]. In productively infected primary cells, flavopiridol displays progressive cytotoxicity [138], as expected for the emergence of TRAP.

Taken together, these observations suggest that TRAP may proceed in productively and latently HIV-infected lymphocytes or macrophages by re-activation of apoptosis via the intrinsic pathway, irrespective of whether the compound is able (CPX, DEF, flavopiridol) or unable (adenine, imatinib) to chelate metal, or is itself a metal chelate (motexafin gadolinium). Such diversity of chemical structures presumably reflects the multiplicity of cascading pathways that converge on mitochondria and trigger the 'altruistic suicide' of pathogenic cells [139], implying that several of these pathways could be targeted for the selective elimination of HIV-1 production sites and sanctuaries. Although therapeutic strategies in HIV/AIDS have to date focused principally on the virus and its immediate cellular targets, requiring continuous administration of suppressive agents, mitochondria are recognized as premier targets for triggering the death of pathogenic cells in oncology, to the point of eradicating the pool of such cells and overcoming the need for long-term medication [139]. It is notable that hydroxyurea (Hydrea™, Droxia™), a pro-apoptotic anticancer drug [140]–[143], suppresses the activity of the HIV-1 promoter [144], depletes HIV-1 DNA in infected cultures thus eliminating off-drug resurgence of the virus [145], and has clinically relevant antiretroviral activity [146], [147] that in some individuals averts viral rebound after drug withdrawal [148]. It has been proposed that this antiretroviral activity of hydroxyurea is not fully explained by the inhibition of its cytostatic target, the R2 subunit of ribonucleotide reductase [149]. Interestingly, the chelating substructure of hydroxyurea is contained within the 1,2-HOPO domain of CPX. We propose that the antiretroviral profile of hydroxyurea is compatible with the concept of TRAP.

### eIF5A and apoptosis of pathogenic cells

CPX and DEF inhibit DOHH, the enzyme responsible for the final step in the formation of hypusine [35], [150]. This posttranslationally modified amino acid has been found exclusively in eIF5A, which in cells exists only in the hypusine-containing form [151]. eIF5A is expressed in lymph nodes during acute HIV-1 infection [152], is overexpressed in lymphocytes of HIV-infected patients [51], and has been implicated as a cofactor for HIV-1 replication [22], [23], [50] as well as a regulator of apoptosis triggered through the intrinsic mitochondrial pathway [47], [48].

Hypusine is essential for eIF5A function in HIV-1 infection and apoptosis. Specifically, a pharmacologically [22], [23], [41]–[46], [52]–[54] or genetically [47]–[49] induced defect of hypusine-containing eIF5A in culture both activates apoptosis in susceptible cells [22], [41]–[48] and inhibits infection by human [22], [23], [49], [52], [53] or feline [54] immunodeficiency virus. Although CPX and DEF could affect other pathways, as discussed below, our data support the view that DOHH and the posttranslational modification of eIF5A are the relevant targets for both drugs. Thus, the inhibition of cellular DOHH by CPX or DEF closely correlates with each agent's antiviral and pro-apoptotic profile (Figs. 4, 5, and S1B) [22], [23]. Notably, the dose-dependencies for TUNEL reactivity and inhibition of eIF5A hydroxylation in CPX-treated H9-HIV cells were indistinguishable (Figs. 4C,D): apoptotic DNA fragmentation correlated positively with the accumulation of non-hydroxylated eIF5A (*r* = +0.995; *P* < 0.01) and negatively with the depletion of hydroxylated eIF5A (*r* = –0.996; *P* < 0.01). Non-hydroxylated precursors of physiologically hydroxylated proteins can antagonize the biological functions of the native protein, as first reported for collagens [153]. Similarly, deoxyhypusyl-eIF5A may interfere with the functions of mature eIF5A and contribute to the induction of apoptosis.

Apoptosis has also been linked to eIF5A hydroxylation in tumor cells that lack functional p53. Like HIV-1 [51], human papillomavirus (HPV) is associated with overexpression of hydroxylated eIF5A [154]. Genetically hypusine-deficient eIF5A causes caspase-dependent apoptosis via the mitochondrial pathway in HPV-immortalized cells [48], which are defective for p53 expression. CPX exerted similar effects in p53-defective tumor cells in culture, and inhibited the growth of breast cancer xenografts in mice by selectively inducing apoptosis in the tumors [155]. This remarkable profile corresponds closely to the pro-apoptotic activity of CPX in HIV-infected cells (Figs. 1, 2, 4 and 6), which in mice leaves the vaginal surface layer of live cells intact despite CPX concentrations in the mM range (Fig. 8). In summary, activation of the intrinsic pathway of apoptosis in HIV- and in HPV-infected cells, triggered by CPX at concentrations that inhibit eIF5A hydroxylation, emerges as a common denominator for the preferential apoptotic elimination of pathogenic, virally infected and/or neoplastic cells in culture and in animals.

### Iron chelation and inhibition of protein hydroxylation

Iron and oxygen availability have been reported to affect HIV-1 infection in a number of studies, implicating several potential mechanisms including cell cycle effects mediated by factors such as eIF5A and CDK2 [156], [157]. Among these, viral transcriptional activation via the cellular kinases CDK2 and CDK9 and HIV-1 Tat has been extensively documented [158]. Iron chelators such as deferasirox (Exjade™) have been shown to inhibit HIV-1 replication via this pathway [159], [160], and a similar mechanism may underlie the inhibition observed with DFOX at supraclinical concentrations [161]–[163]. In our experiments, however, CDK2 is not significantly inhibited by CPX or DEF (Hoque et al., unpublished results) and RNA analysis argues that these chelators inhibit HIV-1 transcription at the level of initiation rather than via the Tat/P-TEFb axis [23]. Furthermore, comparison of CPX and DEF with Agent P2 (at equimolar concentrations to CPX) or DFOX (at clinically relevant concentrations) indicates that metal binding is not sufficient for their antiviral and pro-apoptotic activities or for DOHH inhibition (Figs. 4, 5 and Fig. S1B). We conclude that CPX and DEF do not act exclusively by global depletion of extra- or intracellular iron, by non-specific redox functions of their chelates, or by a broad reduction in metalloenzyme activity, affecting a range of targets for a particular effect [164]. This conclusion is consistent with the observation that protein hydroxylases do not release their non-heme iron center under turnover conditions [165].

All protein hydroxylases display a similarly side chain-coordinated non-heme iron center contained within their active site pocket that is essential for their catalytic utilization of atmospheric oxygen. This event produces an oxo-iron species that inserts its oxygen atom into a specific C-H bond of the substrate protein, as first described [26] and verified [32] for the prolyl 4-hydroxylases. Accordingly, CPX and DEF can also inhibit the non-heme iron oxygenases required for the hydroxylation of collagens [35] and hypoxia-inducible factor (HIF)-1α([166], Hanauske-Abel, unpublished results). Inhibition of collagen hydroxylation decreases its secretion and tissue fibrosis [153], [167]. Fibrosis is a hallmark of HIV-infected lymphoid tissues, limits survival and reconstitution of naïve T cells [168], and predicts the magnitude of the peripheral CD4^+^ cell pool achievable by cART [169]. Adjunctive use of agents that decrease collagen secretion has therefore been proposed to provide antiretroviral benefit [168], [169]. Inhibition of HIF-1αhydroxylation increases its stability and transcriptional activity [170], stimulating the HIV-1 promoter via its specific hypoxia response element [171]. Vpr, itself a pivotal stimulator of HIV-1 expression [78], [122], [124], further increases the level of HIF-1αand enhances HIF-1αbinding to the HIV-1 promoter, augmenting HIV-1 expression [171]. Despite the presence of increased Vpr levels (Fig. 3C), however, both protein hydroxylase inhibitors blocked HIV-1 expression (Fig. 4B).

Our data suggest that CPX interacts with a matching cellular element, the active site cavity of DOHH. Among the iron-binding medicines and experimental compounds surveyed [172]–[175], only CPX and DEF **(i)** comply with the limits for molecular volume and surface area derived from the dimensions of the DOHH substrate, the deoxyhypusine side chain; and **(ii)** meet the requirement for hydrophobic subsite interaction and directional metal-binding imposed by the active site cavity of DOHH (Fig. S3 and [40]). The catalytic iron center of DOHH enables a tertiary structure fold required for substrate binding and product formation, whereas iron removal causes marked refolding with loss of enzyme activity [176]. CPX and DEF render DOHH unable to bind its substrate [23], proposed to reflect their chelating access to the iron center, its release as a chelate from the apoenzyme, tertiary structure collapse, and loss of activity, as evidenced by the accumulation of its substrate, the non-hydroxylated precursor of eIF5A (Fig. 4C and Fig. S1B).

### A mechanistic hypothesis for TRAP and its consequences

**Figure 9:**
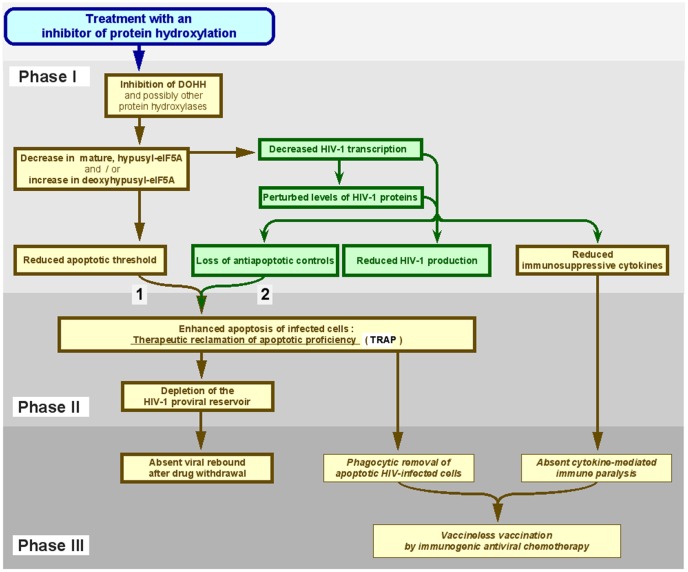
Model for the antiretroviral action of ciclopirox and deferiprone via TRAP. The model is based on two drug effects: (**1**) enhanced pro-apoptotic activity in cells, and (**2**) decreased viral suppression of infection-activated apoptosis due to increased viral pro-apoptotic factors and decreased viral anti-apoptotic factors. In uninfected cells, the apoptotic threshold is decreased (**1**), but they largely escape apoptosis in the absence of infection-activated apoptosis that is released from viral control by drug treatment (**2**). See text for details. Yellow boxes, cellular events; green boxes, viral events. Events measured in this study are specified in bold within bold-lined boxes; predicted consequences are specified in italics within thin-lined boxes.

Based on the data presented here and elsewhere [22], [23], we envision the three-phase model illustrated in Figure 9. In **Phase I**, HIV-1 gene expression is disrupted and production of infective virions and other viral products inhibited, at least in part due to suppression of hypusine formation in eIF5A. These events combine to limit HIV-1 control over the survival of infected cells, leading to TRAP in **Phase II**. The ablation of infected cells diminishes HIV-1 production sites to the point of eradication, evidenced by the lack of HIV-1 resurgence after drug withdrawal in **Phase III**.

In **Phase I**, CPX and DEF inhibit DOHH causing depletion of mature eIF5A and accumulation of its non-hydroxylated precursor (Fig. 4C). This lowers the apoptotic threshold in uninfected cells (Figs. 2A, 6C), consistent with the pro-apoptotic effect of hypusine inhibition. It also blocks HIV-1 gene expression (Fig. 4B; [23]), restricting both productive infection (Fig. 5; Fig. 6A,B) and anti-apoptotic effectors like Tat [63]-[65] (Fig. 3C). Paradoxically, Vpr increases and, consistent with its pro-apoptotic activity [72]-[78], active caspase-3 rises synchronously (Fig. 3C); upon increase of its intracellular concentration, Vpr exerts a proapoptotic instead of an anti-apoptotic function [71]. Vpr can originate from incoming virions [69] and is able to extend its own half-life independent of synthesis by manipulating the ubiquitin/proteasome pathway [70], [125], [177]. This auto-regulatory activity of Vpr, essential for preintegration transcription and initiation of infection [122], [124], [125], [178], is restrained by the subsequent expression of HIV-1 proteins like Vif [179]. We therefore speculate that such feedback control over Vpr is impaired by the drug-mediated suppression of HIV-1 gene expression (Fig. 4B), resulting in an enhancement of Vpr pro-apoptotic activity in HIV-infected cells. Although the intentional recruitment of Vpr as a pharmacological activator of the intrinsic pathway of apoptosis is known to eliminate malignant cells [180], [181], it has not been conceived as a tool for combating HIV-1 infection. Our data suggest that CPX and DEF may recruit Vpr as a caspase-3 activator, facilitating the apoptogenic elimination of virus-infected cells.

In **Phase II**, the drug-induced disruption of eIF5A hydroxylation and HIV-1 gene expression causes TRAP by converting the retroviral blockade of apoptosis (Fig. 1D) into extensive and preferential death of infected cells (Fig. 6C). In our concept, the drug-mediated disruption of viral regulatory mechanisms forces HIV-infected cells over the drug-lowered apoptotic threshold, resulting in depletion of the proviral reservoir. Uninfected cells need to contend solely with a lowered apoptotic threshold. Consequently, in tissue culture the majority of uninfected cells exposed to CPX or DEF survives, in contrast to infected cells (Figs. 1, 2A, 6C and 8; ref. [22]). *In vivo*, non-pathogenic cell populations survive intact even when exposed to drug concentrations that, in the case of CPX, are orders of magnitude above those causing the death of infected cells (Fig. 8). By blocking retroviral gene expression (Fig. 4B; [23]), CPX also quenches the production of host molecules that promote immune paralysis [93], [126], [182]. IL-10 disrupts virus-specific T cell responses and causes persistence of viral infection [183], [184]. This cytokine is induced by several retroviral proteins, e.g. Tat and gp120 [126], [127], [185], is an anti-apoptotic factor for T cells [186] and promotes HIV-1 infectivity through CCR5 induction [187]. The HIV-induced increase in IL-10 levels is not normalized by cART [96] but is completely inhibited by CPX (Fig. 6E).

In **Phase III**, the consequence of suppressed infection in Phase I and of activated apoptosis in Phase II emerges as functional depletion of established virion production sites, evidenced by the absence of post-treatment resurgence (Fig. 7). Resurgence of HIV-1 RNA up to or above the pretreatment set-point occurs in cell culture and in patients after interruption or termination of antiretroviral treatment [188]–[190] and has been attributed to extended survival of HIV-infected cells [191]–[193]. Phase I and Phase II emulate the two major effects of an adaptive immune response, blockade of acute infection (by neutralizing antibodies) and ablation of virally infected cells (by cytotoxic lymphocytes), respectively (see Figs. 5 and 6). The ensuing depletion of viral production sites appears to achieve termination of previously productive infection, suggested by absent resurgence of HIV-1 RNA despite the prolonged post-treatment presence of susceptible cells (Fig. 7 *). In vivo*, absent rebound is a rare event, achieved only by total lymphoid tissue ablation with subsequent transplantation of HIV-1 resistant CCR5▵32/▵32 stem cells [194], [195], by complementing cART with hydroxyurea [148], or by very early start of cART [1]. Importantly, absent re-acquisition of pre-treatment HIV-1 RNA levels was also noted after DEF monotherapy in the proof-of-concept trial (Saxena et al., unpublished data).

### Implications of TRAP: In situ formation of immunogens

In addition to this immunomimetic antiretroviral side activity of CPX and DEF (Figs. 5–7), we anticipate that such medicines may also display immunogenic activity *in vivo.* When introduced into a host with a functioning immune system, HIV-infected PBMCs rendered apoptotic *ex vivo* induce HIV-1 specific cellular and humoral responses that effectively protect against challenge with live HIV-infected cells [196]–[198]. Similarly, infected cells rendered apoptotic *in situ* by medicines like CPX or DEF, might serve as vehicles that deliver retroviral immunogens to an immune system that has, at least temporarily, been de-paralyzed by the same drugs *via* their ability to inhibit HIV-1 gene expression. Such ‘vaccineless vaccination’ would induce a state of stabilized HIV-1 control that extends beyond cessation of treatment and is evidenced by prolonged suppression of HIV-1 RNA. We propose a testable clinical scenario in which an HIV-infected patient receives medication that blocks expression of immunodisruptive viral genes [23] while activating apoptosis of infected cells ([22] and Figs. 1–7), thereby facilitating an immune response against endogenous re-emergence and exogenous re-infection. Conceptually, lack of resurgence *in vivo* may therefore combine drug-mediated apoptotic ablation of viral production sites with a further, post-medication decline in viral load, as noted in the proof-of-concept trial of DEF (Saxena et al., unpublished data). Of note, DEF-treated thalassemic patients display signs of immune activation and are able to launch immune responses against previously unrecognized epitopes [199].

### Implications of TRAP: Drug development and clinical exploration

TRAP offers a novel framework for the development and proof-of-principle testing of antiviral medicines. TRAP bridges the traditional barrier between pharmacological and immunological modes of fighting viral infections. TRAP-triggering low-molecular weight medicines can preferentially kill productively infected lymphocytic cells (Figs. 1, 2, 6; refs. [22], [128], [130]) and macrophages [7], and possibly latently infected cells [137]. TRAP effects can be investigated directly in patients, bypassing the deficiencies of experimental models for human infection, a severe constraint on antiretroviral drug development. Since HIV-1 transmission occurs via mucosal or intravenous routes and particularly affects populations with established indications for administration of CPX or DEF, the TRAP profiles of these drugs can be tested by hypothesis-directed side effect monitoring in HIV-infected patients who use these medicines as directed and indicated for unrelated therapeutic benefit. We envision two populations for such monitoring studies:

1. HIV-positive thalassemic patients who, requiring life-long transfusions and thus exposed to several blood-borne viruses, became iatrogenically infected and are receiving oral DEF (e.g. Ferriprox™) as therapeutically indicated decorporation agent. In resource-limited epidemic settings, HIV-1 infection of such patients is frequent and eventually may affect every fourth child born with thalassemia [200]-[203]. At least one case report on a thalassemic patient suggests that DEF displays antiretroviral activity *in vivo* [204], although the drug had not been prescribed for this purpose.
2. HIV-positive women who suffer from vulvovaginal candidiasis and receive topical CPX (Batrafen™) as therapeutically indicated antifungal agent. Candidiasis causes significant increases of the HIV-1 copy number in cervicovaginal secretions, even in women who test negative for HIV-1 RNA in plasma [205]. Due to the absent induction of resistant yeast species, which sets CPX and its analog rilopirox apart from azole and polyene antifungals [114], [206], [207], and due to its inherent antiretroviral activity, CPX is predicted to suppress vaginal shedding of infectious virions in a manner superior to azoles and polyenes.

Recent studies indicate that CPX may no longer be an exclusively topical medicine. Its systemic application has shown promise in patients with hematologic malignancies, in particular acute myelogenous leukemia [208], [209]. In culture, CPX markedly decreases several proteins that protect malignant cells against apoptosis, among them survivin [210] and Hsp27 (Mémin et al., *submitted*). Both proteins are also induced by HIV-1, ensuring survival of infected cells [211,212]. Our further studies will focus on this convergence in pathogenic, i.e. malignant or HIV-infected cells.

The medicines investigated here as antiretroviral pioneer drugs are widely used and considered safe for their approved human applications. They modulate infection by HIV-1 in culture, and DEF shows activity in infected individuals (Saxena et al., unpublished data). These facts do not, however, elevate these medicines into the category of antiretrovirals: extensive clinical studies have to be completed.

Our data suggest that CPX and DEF can serve as the basic structures for chemical optimizations to achieve TRAP by dedicated cytocidal antivirals. The transformation of the antiviral and pro-apoptotic side activities of existing drugs into the main activity of a cytocidal antiviral can be guided by their steric parameters and structure-activity relation [22], [23] (Fig. 4 and Fig. S3), and by the steric coordinates of their *bona fide* cellular targets, which for CPX and DEF appear to be the active sites and catalytic events of non-heme iron protein hydroxylases [22], [23], [26]–[29], [40].

## Conclusions

Two structurally distinct drugs, the antifungal ciclopirox and the chelator deferiprone, inhibit HIV-1 gene expression and activate the intrinsic pathway of apoptosis preferentially in infected cells. In contrast to current antiretrovirals, these medications therefore terminate the infection by HIV-1 of human lymphocytes in culture. This finding suggests a general strategy for combating HIV/AIDS and potentially other infections: the therapeutic reclamation of apoptotic proficiency.

## Materials and Methods

### CPX, DEF and DFOX

CPX, as its mono-ethanolammonium salt (‘ciclopirox olamine’), was obtained from Sigma Chemical Co., St Louis, MO. Dissolved in sterile, trace metal-free Earle’s Solution (Sigma Chemical Co., St Louis, MO), 20 mM stock solutions were maintained at 4 °C, used for four weeks, and then discarded. Stock solutions were not frozen. In solutions containing trace metals and phosphate, CPX may form a faint precipitate that renders their use unreliable: verification of absent CPX precipitates is essential. For mouse studies, 1% Batrafen Vaginalcrème™, an oil-in-water preparation containing 28.8 mM total and 0.6 mM bioavailable CPX, was obtained from Sanofi-Aventis, Frankfurt, Germany. Drug-grade DEF was provided by Apotex (Toronto, Canada) and DFOX was purchased from Sigma Chemical Co. Stock solutions (20 mM and 2 mM, respectively) were prepared and handled as above. CPX and DEF were used at 30 µM and 200 µM, respectively, except where otherwise specified.

### Synthesis of Agent P2 (1-hydroxy-4-methylpyridin-2[1H]-one)

4-picoline N-oxide (1.14 g, 10.43 mmol) in tetrahydrofuran (54 ml, distilled from sodium/benzophenone under N_2_) was cooled to –78°C in a dry ice-acetone bath. *n*-Butyllithium (1.6 M in hexanes, 13.0 ml, 20.8 mmol) was added, the red-brown mixture stirred for 1 hr under nitrogen and then oxygen-bubbled for 30 min. Brought to room temperature, water (30 ml) was added, the mixture acidified to pH 2 with hydrochloric acid and extracted with chloroform (8×55 ml). The extracts were dried with Na_2_SO_4_, filtered, and the filtrate evaporated under reduced pressure. A yellow-brown residue was purified by chromatography (silica gel, ether) yielding 1-hydroxy-4-methylpyridin-2[1H]-one [213] by CHN analysis and the following criteria: 1H NMR (300 MHz, CDCl_3_, δ): 10.18 (1H, br, OH); 7.63 (1 H, d, J = 7 Hz, aromatic); 6.49 (1 H, d, J = 2 Hz, aromatic); 6.15 (1 H, dd, J = 7 Hz, 2 Hz, aromatic); 2.22 (3 H, s, CH_3_). MS(EI): *m/z* 125 [M^+^.].

### H9 and H9-HIV cell lines

Uninfected and uniformly HIV-1 (HTLV-IIIB) infected H9 cells, obtained from the NIH AIDS Research and References Reagent Program, were cultured at 37°C in a humidified atmosphere (5% CO_2_, 95% humidity) using RPMI 1640 medium supplemented with 2 mM L-glutamine, 100 µg/ml penicillin, 100 µg/ml streptomycin and 20% fetal calf serum.

### Uninfected PBMCs

Using an IRB-approved protocol, PBMCs were isolated from the blood of healthy donors and stimulated overnight with phytohemagglutinin (PHA) and human IL-2 [214]. Stimulated cells were pelleted and resuspended for culture at a final concentration of 5×10^5^ cells/ml in PHA-free RPMI 1640 medium containing 10% fetal calf serum (v/v), 100 units/ml penicillin G, 100 µg/ml streptomycin, 2 mM glutamate, and 3.5 ng/ml human IL-2 (Medium B). Cultures were incubated at 37 °C, 5% CO_2_, and 95% humidity.

### Infectious virus stock

Using an IRB-approved protocol, two donors were recruited to generate clinical viral isolates. One (#990,135) was highly immunocompromised despite on-going cART (CD4 count < 5%; HIV RNA in plasma at log_10_ 5.5 copies/ml). The second (#990,010) was moderately to severely immunocompromised on cART (CD4 count 14 – 26%; HIV RNA in plasma at log_10_ 3.8 – 5.0 copies/ml). For infection, 5×10^6^ uninfected stimulated PBMCs were co-cultured with 1×10^7^ PBMCs from one of the HIV-infected donors in Medium B. On day 3, half of the supernatant was removed and replenished with an equal volume of fresh Medium B. On day 7, the medium was likewise replenished and 7.5×10^6^ stimulated uninfected PBMCs were added. On days 10, 17, and 24, half of the supernatant was replenished. On days 14, 21, and 28, stimulated uninfected PBMCs were added. Cells were harvested when p24 reached 250 pg/ml, cryopreserved in freezing medium (90% fetal calf serum, 10% dimethyl sulfoxide), and stored in liquid nitrogen as infected PBMC stock. Cell-free supernatants were stored at –80 °C.

### Acute PBMC infection model

Uninfected PBMCs were co-incubated with infected PBMC stock in 24-well microplates at 2 ml/well Medium B, at a 10∶1 ratio of uninfected-to-infected cells, and a total cell number of 1×10^6^. Cultures were maintained and assayed for 6 consecutive days. CPX or Agent P2 was added at the time of inoculation or 12 hr later. Leaving the cell layer undisturbed, half the medium in each well was replenished every day, with concurrent adjustment of compound concentration. On each day during the 6-day experiments, a set of wells was harvested: the cells were processed for determination of viability and apoptosis, and the cell-free supernatants were stored at –80°C for p24 and viral RNA measurements. Experiments were performed at least in duplicate and repeated at least twice.

### Persistent PBMC infection model

Uninfected (5×10^6^ cells) and infected PBMCs (5×10^5^ cells) were co-incubated in a 25 ml culture flask at a final concentration of 2.2×10^5^ cells/ml. Cultures were allowed to establish productive infection, defined by medium p24 above 250 pg/ml, and CPX was added. Cultures were replenished with Medium B and freshly isolated uninfected PBMCs on alternate days. For replacement of Medium B, half of the supernatant was gently exchanged without disturbing the cells, and the drug concentration was adjusted appropriately. For replacement with freshly isolated, stimulated and uninfected PBMCs, half of the cells and supernatant were removed and replaced with 2.5×10^6^ cells in the proper volume of Medium B, with adjustment of the drug concentration. Cell-free supernatants were saved for p24 and viral RNA measurements.

### Quantitation of cell number, viability, and diameter

Viability, diameter, and cell number of PBMCs and H9 cells were measured by computerized image analysis of trypan blue exclusion (VI-CELL™; Beckman Coulter; Fullerton, CA). PBMCs were counted with an automated, multi-parameter hematology analyzer (CELL-DYN™; Abbott Laboratories, Abbott Park, IL).

### Mitochondrial membrane potential (▵Ψ) and DNA fragmentation assays

The potential across the mitochondrial membrane of live cells was determined flow-cytometrically with the lipophilic cationic fluorochrome JC-1 (5,5′,6,6′-tetrachloro-1,1′,3,3′-tetraethylbenzimidazolcarbocyanine iodide) immediately after sample harvest (BD™ Mitoscreen Kit, BD Biosciences; San Diego, CA). DNA fragmentation was quantified flow-cytometrically, using a TUNEL (terminal deoxynucleotide transferase dUTP nick end-labeling) assay (APO-BRDU™; Phoenix Flow Systems; San Diego, CA) and a dual-color assay for annexin V binding and 7-AAD exclusion (Annexin V-PE 7-AAD Apoptosis Detection Kit I™; BD Biosciences Pharmingen, San Jose, CA). The apoptotic volume decrease of cells was assessed by the reduction of their diameter (VI-CELL™; Beckman Coulter; Fullerton, CA).

### Quantitation of intracellular proteins

Intracellular antigens were determined, as geometric means of fluorescence, by flow cytometric analysis using a BD FACSCalibur™ and its BD FACStation System™ (Becton Dickinson; San Jose, CA). Intracellular antigens were stained according to the instrument producer’s instructions. Anti-HIV-1 Tat antibody was from Abcam Inc. (Cambridge, MA); anti- HIV-1 Vpr from Santa Cruz Biotechnology (Santa Cruz, CA); anti-HIV-1 Rev from Advanced Biotechnologies Inc. (Columbia, MD); anti-HIV-1 p24 (KC57) from Beckman Coulter, Miami, FL; monoclonal C92-605 anti-human caspase-3 (active form) from Pharmingen (BD Biosciences, San Diego, CA); monoclonal F21-852 anti-human poly (ADP-ribose) polymerase (caspase-cleaved 89-kDa fragment); and monoclonal 6C8 anti-human Bcl-2 from Becton Dickinson (BD Biosciences; San Diego, CA).

### Gene expression assays

293T cells (1×10^5^ cells/well of 12-well microplates) were seeded and transfected 20 hr later with a firefly luciferase (Luc)-expressing reporter plasmid and corresponding *Renilla* luciferase (Ren)-expressing reference plasmid using TransFectin™ (Bio-Rad, Hercules, CA). The chelatable intracellular iron concentration was monitored using plasmids obtained from B. Galy and M.W. Hentze (EMBL, Heidelberg). Plasmid pcFIF-Luc contains the mouse ferritin H 5’-UTR with an IRE controlling Luc; pcFIRo-Ren is a similar construct expressing a mutated non-functional IRE fused to Ren. The activity of the HIV-1 promoter was monitored using the pNL4-3-Luc E^−^ molecular clone [215], based on the recombinant infectious proviral clone that contains DNA from HIV isolates NY5 (5' half) and IIIB (3' half). This plasmid, obtained from D. Baltimore (Caltech, Pasadena), contains the Luc gene in place of 102 nucleotides from *nef* and 6 nucleotides from *env.* pCMV-Ren (Promega, Madison, WI) was used as reference for pNL4-3-Luc E^−^. Compounds were added at the time of transfection. Cells were harvested 12 hr later, washed with phosphate-buffered saline, and lysed for the dual luciferase assay (Dual Luciferase™ Reporter Assay System; Promega, Madison, WI). Luc data were normalized to the Ren internal controls [23].

### DOHH assay

Peptide-bound hypusine and deoxyhypusine were measured as described [40].

### Quantitation of p24, viral copy number, and cytokines

p24 core antigen in the supernatant was quantified by ELISA (Retrotek HIV-1 p24™; ZeptoMetrix Corp.; Buffalo, NY). HIV-1 RNA copy number in the supernatant of cell cultures and in plasma of patients enrolled in the exploratory DEF trial was determined with a PCR-based and FDA-approved assay (Amplicor HIV-1 Monitor™; Roche Diagnostics Corp.; Indianapolis, IN). IFN-γ and IL-10 in the supernatant were determined by cytometric bead array (Human Th1/Th2 Cytokine Kit II™; BD Biosciences Pharmingen, San Jose, CA).

### Mouse model for cervicovaginal toxicity of CPX

As described previously [111], 10 week old female CF-1 mice (Harlan; Indianapolis, IN) received 2.5 mg medroxyprogesterone acetate (MPA) administered subcutaneously to induce superficial mucification of the vagina, i.e. a surface layer of vital columnar instead of dead cornified cells. Five days after injection, three groups of ten animals each were formed. Animals in Groups A and B received 20 µl 1% Batrafen Vaginalcrème™ containing 28.8 mM total and 0.6 mM bioavailable CPX on four consecutive days. Animals in Group C (controls) received 20 µl phosphate buffered saline on four consecutive days. All animals were then challenged by vaginal inoculation with HSV-2, the animals in Groups A and C receiving high-dose (10 ID_50_) and those in Group B low-dose (0.1 ID_50_) inoculum. Vaginal lavages from all animals were obtained three days after inoculation and assayed for viral shedding using an anti-HSV-2 FITC-conjugated mouse monoclonal antibody for direct fluorescence detection of HSV-2 antigen expression (Bartels™ Herpes Simplex Virus Type-Specific Fluorescent Monoclonal Antibody Test; Trinity Biotech, Bray, Ireland) in cell cultures inoculated with the vaginal lavages. Bright green fluorescence of the inoculated wells was read as an HSV-2 - positive reaction. In this murine model of vaginal susceptibility to infection *in situ*, the high-dose inoculum infects on average 87% and the low-dose inoculum 13% of untreated control animals [111]. To assess the histological effect of this drug regimen on the vaginal mucosa, two test animals pretreated with MPA received 20 µl Batrafen for three consecutive days, two control animals pretreated with MPA received no further treatment, and two additional control animals were untreated. On the third day, 2.5 hours after delivering the third dose of Batrafen Vaginalcrème™ to the test animals, all six animals were humanely sacrificed, the entire genital tract dissected as a single-organ package, fixed in 10% neutral-buffered formalin, paraffin-embedded, sectioned, and stained/counterstained with hematoxylin/eosin. To visualize apoptotic cells in the reproductive tract tissues of untreated and Batrafen-treated mice, sections were stained for the active form of caspase-3 using monoclonal antibody C92-605. This reagent is specific for active caspase-3 and lacks cross-reactivity against pro-caspase-3. Signal generation required the application of a cycled microwave irradiation protocol for antigen retrieval, using a commercially available buffer system (Citra™, BioGenex; San Ramon, CA). Immunostaining was optimal at a dilution of 1∶125 after overnight incubation. In identically prepared sections of the same tissue blocks that were to be evaluated as negative controls, the anti-human caspase-3 (active form) reagent was omitted. For signal generation, the streptavidin-biotin/horseradish peroxidase complex technique was used, with diaminobenzidine as chromogen and hematoxylin as counterstain. Endogenous peroxidase was blocked in all tissue sections as described [154]. All slides were examined in a blinded manner by an experienced pathologist specialized in the analysis of human and rodent female genital tract histology.

### Data analysis

For the *in vitro* and cell culture experiments with CPX and DEF, descriptive statistics were generated using Microsoft Excel 2011. In the flow cytometry-based protein quantifications, the mean ± SEM were calculated for the geometric means of fluorescence and the ratio of control vs. treated cells was compared with the SEM in a t-test to assess the change relative to the precision of measurement. Time course of cytokines was correlated with the time course of viral parameters. Statistical significance was based on the number of measurements per correlation.

### Ethics statement

**a.** The human protocol for the primary cell culture experiments was conducted at the New Jersey Medical School, University of Medicine and Dentistry of New Jersey (now Rutgers University), Newark, NJ, USA, and covered the isolation and handling of mononuclear cells from peripheral blood of HIV-infected and uninfected volunteers. Blood draws from an antecubital vein for the purposes of this study were approved by the Institutional Review Board of the University of Medicine and Dentistry, Newark, NJ (Protocol #0119990009) and performed only after obtaining written informed consent by study participants.

**b.** The animal protocol was conducted at Johns Hopkins University, Baltimore, MD, in facilities administered by the university's program for laboratory animals. This program for the humane and scientifically appropriate care of laboratory animals is accredited by the Association for the Assessment and Accreditation of Laboratory Animal Care International (AAALAC) in facilities that comply with applicable regulatory guidelines set forth in the Animal Welfare Act regulations of the U.S. Department of Agriculture (7 U.S.C. 2132 *et seq.*) per Animal Assurance Number A-3272-01. Experiments were conducted in strict accordance with the recommendations in the *Guide for the Care and Use of Laboratory Animals* of the Institute of Laboratory Animal Resources, Commission on Life Sciences, National Research Council. Experiments were conducted in accordance with a protocol approved by the University's IACUC (Protocol Number MO05A302, P.I. Dr. Cone). The method used for euthanasia complied with the recommendations of the university's Animal Use Committee and the Panel on Euthanasia of the American Veterinary Medical Association. All efforts were made to minimize distress and suffering.

212Wainberg Z, Oliveira M, Lerner S, Tao Y, Brenner BG (1997) Modulation of stress protein (hsp27 and hsp70) expression in CD4+ lymphocytic cells following acute infection with human immunodeficiency virus type-1. Virology 233: 364–373.

213. Abramovitch RA, Knaus EE (1975) The direct thionation and aminoalkylation of pyridine 1-oxides and related reaction. J Heterocycl Chem 12: 683–690.

## Supporting Information

Figure S1 **Characteristics of Ciclopirox and Agent P2. A. Interaction with iron.** UV-visible absorption spectra of CPX (*blue*), Agent P2 (*cyan*), and ferric chloride (Fe ^3+^; *green*), and of the CPX-Fe ^3+^ and Agent P2-Fe ^3+^ complexes in Earle’s Solution, pH 7.3. Data was acquired using a UV-visible Cary Bio 100 spectrophotometer and WinUV software (Varian, Walnut Creek, CA). CPX-Fe^3+^ and the P2-Fe^3+^ solutions both generated maximum absorption at 410 nm, showing that they form identical bidentate tris(N-hydroxypyridinone ligand) complexes [218]. The CPX-iron complex contains three hydrophobic cyclohexyl moieties and tends to precipitate out of solution, in contrast to the Agent P2-iron complex. The iron complexes of the medicinal chelator DFOX, but not DFOX itself, likewise display a maximum at 410 nm ([218]; data not shown). **B**. **Dose-dependant inhibition of DOHH activity** ***in vitro*** **by CPX (blue) and its chelation homolog Agent P2 (cyan).** The partially purified rat DOHH enzyme had a maximal activity of 2.3 pmol/mg/hr and was employed at 0.44 mg total protein per reaction.
(TIF)

Figure S2 **Analysis of the dilutional effect of the replenishment protocol on retroviral copy number in the long-term PBMC cultures.** We computationally analyzed the potentially confounding effect of the replenishment protocol developed for the long-term PBMC cultures on the results obtained in the long-term PBMC cultures infected with patient isolates. On alternate days, the cultures were replenished with medium and PBMCs freshly isolated from HIV-uninfected HLA-diverse volunteers. Replenishment of these multi-donor mixed lymphocyte cultures was required to meet cell decay and nutritional demands in the productively infected controls. The cultures generated extraordinarily high levels of infectious HIV (≥10^6^ copies/ml) that have only been observed in moribund AIDS patients. Cell proliferation kinetics in mixed lymphocyte cultures from two HLA-diverse individuals are complex due to differential survival of stimulator/responder and daughter subpopulations, the modifying impact of initial cell number, etc. In our system, this complexity is multiplied by the necessity to rely on PBMCs from *multiple* HLA -diverse individuals and by the presence of both a viral infection and a drug. We do not know of any methodology that could deconvolute the differential survival and kinetics of the multitudes of subpopulations in these cultures. Viral propagation is the key parameter of our study, however, and the impact of replenishment can be assessed mathematically at the level of HIV-1 copy number. Our model is based on the replenishment parameters (frequency, volume, etc.) and the observation that 30 µM CPX (the concentration maintained throughout the 38-day treatment period) completely blocks virion generation in infected PBMCs (Figs. 5B and 6B). The viral copy number at the start of CPX treatment is therefore ‘frozen’ and subject only to change by dilution and decay. Ignoring decline by decay, change by dilution can be calculated and represents a high-end estimate for the fall in virion number. This computational model indicates that medium replenishment by itself (in the absence of viral replication and decay) will dilute viral copy number from over 2×10^6^ copies/ml on day 1 of CPX to the limit of HIV-1 RNA detectability (about 200 copies/ml) by day 23 and to less than 0.01 copies/ml on day 38 of CPX (shown in blue). The experimentally determined virion numbers (shown in red) deviate markedly from this model. Within the first days on CPX, a gap that widens over time places the measurements significantly *above*, but not at or below, those modeled as representing the dilutional decline in viral copies. Thus, the replenishment protocol does not by itself result in a dilution of the virus sufficient to cause lack of HIV-1 rebound after drug withdrawal.
(TIF)

Figure S3 **Inhibitor domains and active site organization of DOHH.** **A. Computationally derived geometric dimensions of peptide-bound hypusine, ciclopirox and Agent P2.** The oxygen atom at C9 of hypusine reveals the position of the non-heme iron (blue circle) that is essential for its generation and for coordination of the inhibitors. The deoxyhypusine substrate of DOHH displays the same conformation as hypusine, but lacks the oxygen atom at C9 (not shown). Molecules are depicted as tube models in energetically minimized conformation and conventionally colored (*gray*, carbon; *red*, oxygen; *blue*, nitrogen; *white*, hydrogen). The green double arrows specify the largest intercarbon distance within the hydrophobic substructures of the hypusine residue and ciclopirox. The van der Waals-based cavitand contour around ciclopirox approximates the van der Waals-based shape of the hypusine residue and fits the proposed dimensions of the active site pocket of DOHH [40]. White arrows identify oxygen atom-mediated bidentate chelation. Within the active site cavity of DOHH, the hydrophobic cyclohexyl moiety of CPX locates to the site of the hydrophobic (CH_2_)_4_ – segment in the lysyl domain of the substrate. The scheme on the right indicates that ciclopirox shares two functionally discernable domains, one for hydrophobic anchorage (green) and one for coordinative anchorage (blue), whose alignment meets the directional metal-binding requirements imposed by the active site cavity of DOHH [40]. Only the coordinative domain occurs in the chelation homolog Agent P2, which despite the electronically and spectroscopically identical metal binding moiety (Figs. 4A and S1A) is distinctly less inhibitory for DOHH *in vitro* (Fig. S1B). Only the stereochemistry of the CPX domains for hydrophobic (green) and for coordinative anchorage (blue) conforms with the experimentally derived model for the active site architecture of DOHH [40]. **B. Computational analysis of CPX, Agent P2, DEF, and the DOHH substrate side chain.** Steric parameters were calculated with Spartan™ (Wavefunction Inc., Irvine, Ca). Surface area, volume, and surface area/volume ratio of CPX differ from those of the deoxyhypusine substrate by ≤4 %, whereas those of Agent P2 differ by as much as 47 %.
(TIF)
