# Supplementary figures and images for: Drug-Induced Reactivation of Apoptosis Abrogates HIV-1 Infection
Source: PLoS One. 2013 Sep 23;8(9):e74414. doi: 10.1371/journal.pone.0074414 (PMC3781084; doi:10.1371/journal.pone.0074414)

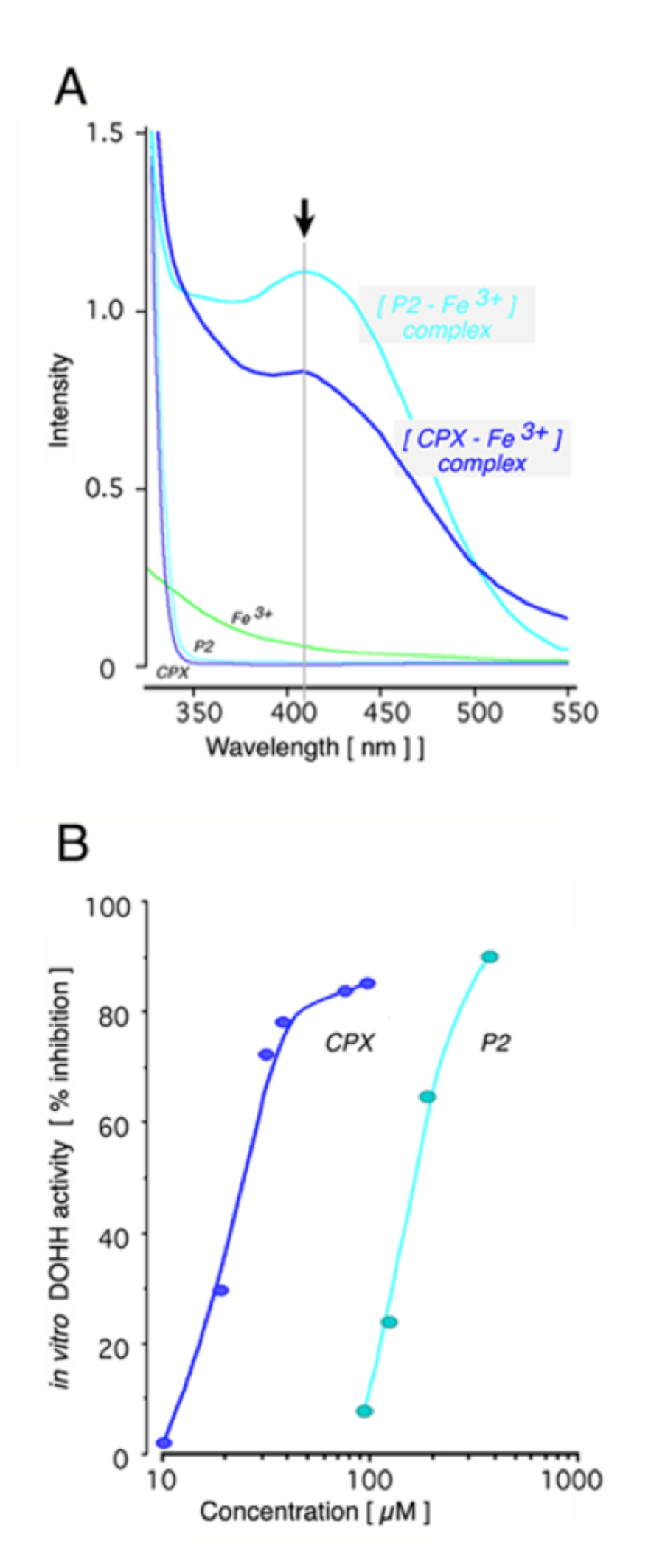

Supplement: Figure S1 — Characteristics of Ciclopirox and Agent P2. A. Interaction with iron. UV-visible absorption spectra of CPX (blue), Agent P2 (cyan), and ferric chloride (Fe 3+; green), and of the CPX-Fe 3+ and Agent P2-Fe 3+ complexes in Earle’s Solution, pH 7.3. Data was acquired using a UV-visible Cary Bio 100 spectrophotometer and WinUV software (Varian, Walnut Creek, CA). CPX-Fe3+ and the P2-Fe3+ solutions both generated maximum absorption at 410 nm, showing that they form identical bidentate tris(N-hydroxypyridinone ligand) complexes [218]. The CPX-iron complex contains three hydrophobic cyclohexyl moieties and tends to precipitate out of solution, in contrast to the Agent P2-iron complex. The iron complexes of the medicinal chelator DFOX, but not DFOX itself, likewise display a maximum at 410 nm ([218]; data not shown). B. Dose-dependant inhibition of DOHH activity in vitro by CPX (blue) and its chelation homolog Agent P2 (cyan). The partially purified rat DOHH enzyme had a maximal activity of 2.3 pmol/mg/hr and was employed at 0.44 mg total protein per reaction. (TIF) [file pone.0074414.s001.tif]

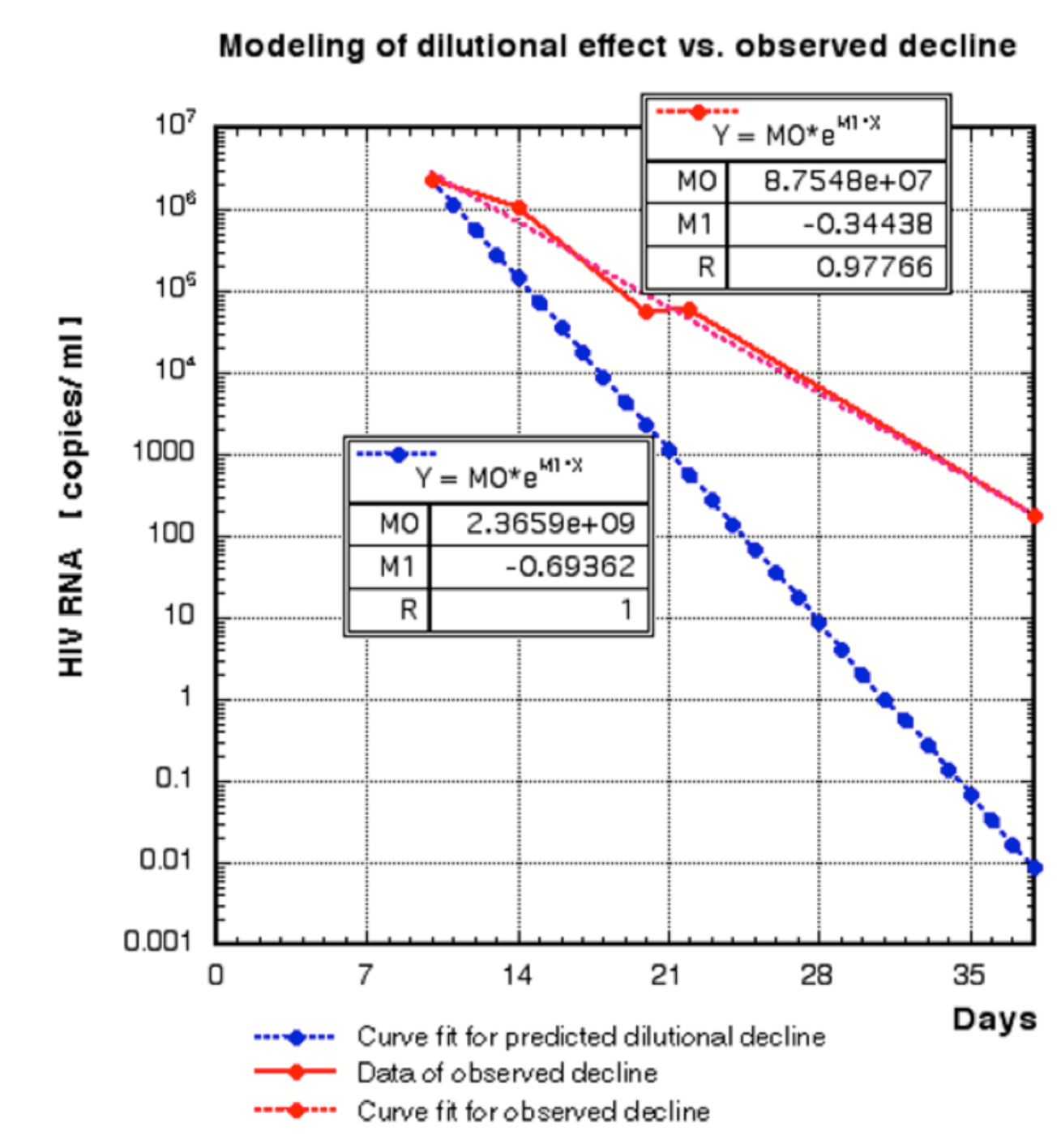

Supplement: Figure S2 — Analysis of the dilutional effect of the replenishment protocol on retroviral copy number in the long-term PBMC cultures. We computationally analyzed the potentially confounding effect of the replenishment protocol developed for the long-term PBMC cultures on the results obtained in the long-term PBMC cultures infected with patient isolates. On alternate days, the cultures were replenished with medium and PBMCs freshly isolated from HIV-uninfected HLA-diverse volunteers. Replenishment of these multi-donor mixed lymphocyte cultures was required to meet cell decay and nutritional demands in the productively infected controls. The cultures generated extraordinarily high levels of infectious HIV (≥106 copies/ml) that have only been observed in moribund AIDS patients. Cell proliferation kinetics in mixed lymphocyte cultures from two HLA-diverse individuals are complex due to differential survival of stimulator/responder and daughter subpopulations, the modifying impact of initial cell number, etc. In our system, this complexity is multiplied by the necessity to rely on PBMCs from multiple HLA -diverse individuals and by the presence of both a viral infection and a drug. We do not know of any methodology that could deconvolute the differential survival and kinetics of the multitudes of subpopulations in these cultures. Viral propagation is the key parameter of our study, however, and the impact of replenishment can be assessed mathematically at the level of HIV-1 copy number. Our model is based on the replenishment parameters (frequency, volume, etc.) and the observation that 30 µM CPX (the concentration maintained throughout the 38-day treatment period) completely blocks virion generation in infected PBMCs (Figs. 5B and 6B). The viral copy number at the start of CPX treatment is therefore ‘frozen’ and subject only to change by dilution and decay. Ignoring decline by decay, change by dilution can be calculated and represents a high-end estimate for the fall in virion numb [file pone.0074414.s002.tif]

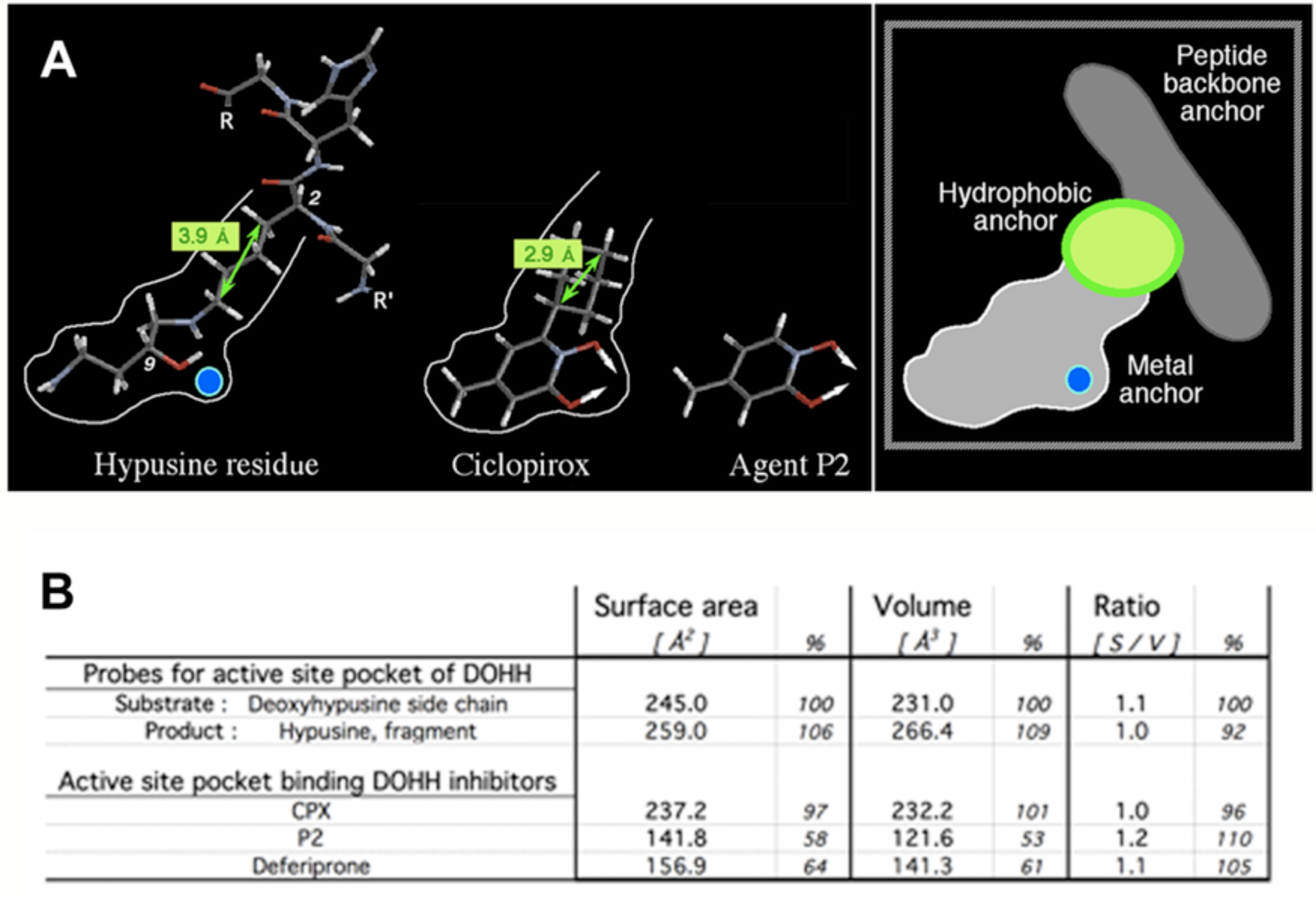

Supplement: Figure S3 — Inhibitor domains and active site organization of DOHH. A. Computationally derived geometric dimensions of peptide-bound hypusine, ciclopirox and Agent P2. The oxygen atom at C9 of hypusine reveals the position of the non-heme iron (blue circle) that is essential for its generation and for coordination of the inhibitors. The deoxyhypusine substrate of DOHH displays the same conformation as hypusine, but lacks the oxygen atom at C9 (not shown). Molecules are depicted as tube models in energetically minimized conformation and conventionally colored (gray, carbon; red, oxygen; blue, nitrogen; white, hydrogen). The green double arrows specify the largest intercarbon distance within the hydrophobic substructures of the hypusine residue and ciclopirox. The van der Waals-based cavitand contour around ciclopirox approximates the van der Waals-based shape of the hypusine residue and fits the proposed dimensions of the active site pocket of DOHH [40]. White arrows identify oxygen atom-mediated bidentate chelation. Within the active site cavity of DOHH, the hydrophobic cyclohexyl moiety of CPX locates to the site of the hydrophobic (CH2)4 – segment in the lysyl domain of the substrate. The scheme on the right indicates that ciclopirox shares two functionally discernable domains, one for hydrophobic anchorage (green) and one for coordinative anchorage (blue), whose alignment meets the directional metal-binding requirements imposed by the active site cavity of DOHH [40]. Only the coordinative domain occurs in the chelation homolog Agent P2, which despite the electronically and spectroscopically identical metal binding moiety (Figs. 4A and S1A) is distinctly less inhibitory for DOHH in vitro (Fig. S1B). Only the stereochemistry of the CPX domains for hydrophobic (green) and for coordinative anchorage (blue) conforms with the experimentally derived model for the active site architecture of DOHH [40]. B. Computational analysis of CPX, Agent P2, DEF, and the DOHH substrate side chai [file pone.0074414.s003.tif]
